# Artificial Macrophage with Hierarchical Nanostructure for Biomimetic Reconstruction of Antitumor Immunity

**DOI:** 10.1007/s40820-023-01193-4

**Published:** 2023-09-22

**Authors:** Henan Zhao, Renyu Liu, Liqiang Wang, Feiying Tang, Wansong Chen, You-Nian Liu

**Affiliations:** 1 Hunan Provincial Key Laboratory of Micro & Nano Materials Interface Science, College of Chemistry and Chemical Engineering, Central South University, Changsha, 410083 Hunan People’s Republic of China; 2 Xiangya Hospital, Central South University, Changsha, 410008 Hunan People’s Republic of China; 3 Henan Province Industrial Technology Research Institute of Resources and Materials, School of Material Science and Engineering, Zhengzhou University, Zhengzhou, 450001 Henan People’s Republic of China; 4 College of Chemical Engineering, Xiangtan University, Xiangtan, 411105 Hunan People’s Republic of China

**Keywords:** Artificial macrophage, Chemical messenger, Hierarchical nanostructure, Anoikis, Antitumor immunotherapy

## Abstract

Artificial cells are constructed from synthetic materials to imitate the biological functions of natural cells. By virtue of nanoengineering techniques, artificial cells with designed biomimetic functions provide alternatives to natural cells, showing vast potential for biomedical applications. Especially in cancer treatment, the deficiency of immunoactive macrophages results in tumor progression and immune resistance. To overcome the limitation, a BaSO_4_@ZIF-8/transferrin (TRF) nanomacrophage (NMΦ) is herein constructed as an alternative to immunoactive macrophages. Alike to natural immunoactive macrophages, NMΦ is stably retained in tumors through the specific affinity of TRF to tumor cells. Zn^2+^ as an “artificial cytokine” is then released from the ZIF-8 layer of NMΦ under tumor microenvironment. Similar as proinflammatory cytokines, Zn^2+^ can trigger cell anoikis to expose tumor antigens, which are selectively captured by the BaSO_4_ cavities. Therefore, the hierarchical nanostructure of NMΦs allows them to mediate immunogenic death of tumor cells and subsequent antigen capture for T cell activation to fabricate long-term antitumor immunity. As a proof-of-concept, the NMΦ mimics the biological functions of macrophage, including tumor residence, cytokine release, antigen capture and immune activation, which is hopeful to provide a paradigm for the design and biomedical applications of artificial cells.

**Supplementary Information:**

The online version contains supplementary material available at 10.1007/s40820-023-01193-4.

## Highlights

- An artificial macrophage with hierarchical nanostructure (BaSO_4_@ZIF-8/TRF NMΦ) is constructed as an alternative to immunoactive macrophages.
- The Zn^2+^ chemical messenger as an “artificial cytokine” is released from the artificial macrophage to induce tumor anoikis and enhance immunogenicity.
- The artificial macrophage can efficiently capture tumor antigens for antigen presentation and T cell activation to fabricate long-term antitumor immunity, successfully mimicking the basic functions of natural immunoactive macrophage.

**Supplementary Information:**

The online version contains supplementary material available at 10.1007/s40820-023-01193-4.

## Graphical abstract

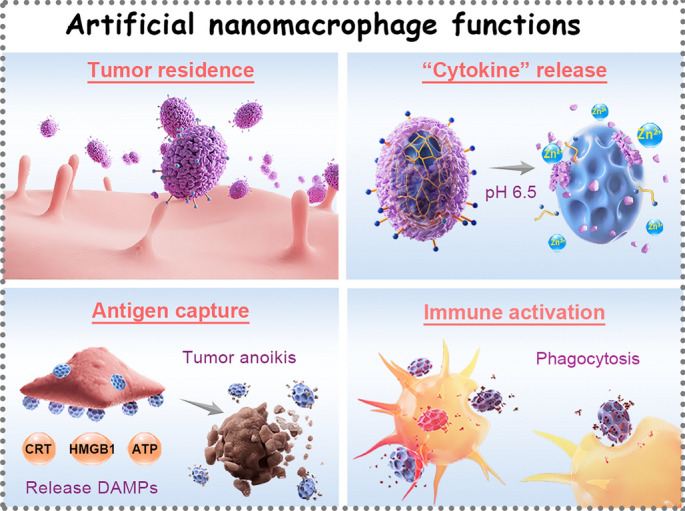

## Introduction

Biomimetics utilizes synthetic materials to mimic the biological functions or processes in nature, which has attracted interdisciplinary research interest [1–3]. As a typical example of biomimetics, artificial cells are constructed from synthetic materials to imitate all or part of the biological functions of natural cells [4]. The synthetic and tunable structures of artificial cells allow them to adequately exert various physicochemical interactions with cells [5]. Moreover, the functions of artificial cells can be flexibly designed and adjusted according to practical requirements [6]. Besides, artificial cells are facile to be produced on a large scale with low cost [7]. All these merits make artificial cells a promising alternative to natural cells in the treatment of diseases [8]. Especially in cancer immunity, the deficiency of natural immunoactive macrophages gives rise to tumor progression and immune resistance [9]. Moreover, the residence of immunosuppressive macrophages impairs the infiltration of T cells and drives T cell exhaustion in tumors via the secretion of anti-inflammatory cytokines [10, 11]. As a result, neither innate nor adaptive antitumor immunity can be activated to defend against cancer [12, 13]. To supplement immunoactive macrophages, researchers have paid tremendous endeavors to develop chimeric antigen receptor (CAR) cells via cell engineering, such as CAR-macrophages [14]. CAR cells are engineered to express tumor-specific receptors on immune cells by gene editing technology, but its application is restricted by elaborate construction, high cost and tumor heterogeneity [15–17]. Thus, developing artificial macrophages as an alternative to immunoactive macrophages would be more preferable for cancer therapy.

To date, artificial macrophages have been rarely fabricated to mimic natural macrophages, probably due to the following challenges [18]. Firstly, the biological functions of natural macrophages highly rely on the pro-inflammatory cytokines, such as tumor necrosis factor α (TNF-α) and interleukins (ILs), which can mediate tumor apoptosis and antitumor immunity [19, 20]. The ability of releasing cytokines to initiate immunity is a prerequisite for the design of artificial macrophages. Second, antigen capture is a key pivotal step for natural macrophages in mediating antigen presentation and T cell activation [21]. In most cases, antigen capture is accomplished by natural macrophages via nonspecific phagocytosis [22]. However, few artificial macrophages have been successfully constructed to fulfill the above requirements.

With the advances in nanotechnology, nanomaterials have been developed to mimic versatile cellular functions [23, 24]. Especially, nanomaterials with the component of chemical messengers, such as Ca^2+^, Mg^2+^, Zn^2+^ and H_2_S, are highly effective in regulating physiological processes and cellular communication [25]. The trace variation of chemical messengers may play regulatory roles in either antitumor therapy or immune activation, similar to those of pro-inflammatory cytokines [26]. For example, intracellular Ca^2+^ or Zn^2+^ overload can effectively impede tumor growth, and activate antitumor specific immune response to arrest tumor metastasis [27, 28]. Given the pivotal roles in immune regulation, chemical messengers are expected to be “artificial cytokines” of the synthetic macrophages. Besides, nanomaterials have been utilized as nanocarriers for the transfer of bioactive cargoes, such as drugs, enzymes, genes, and even antigens [29]. Nanomaterials with high loading capacity lay a foundation for the antigen capture of nanoscaled artificial macrophages.

**Fig. 1:**
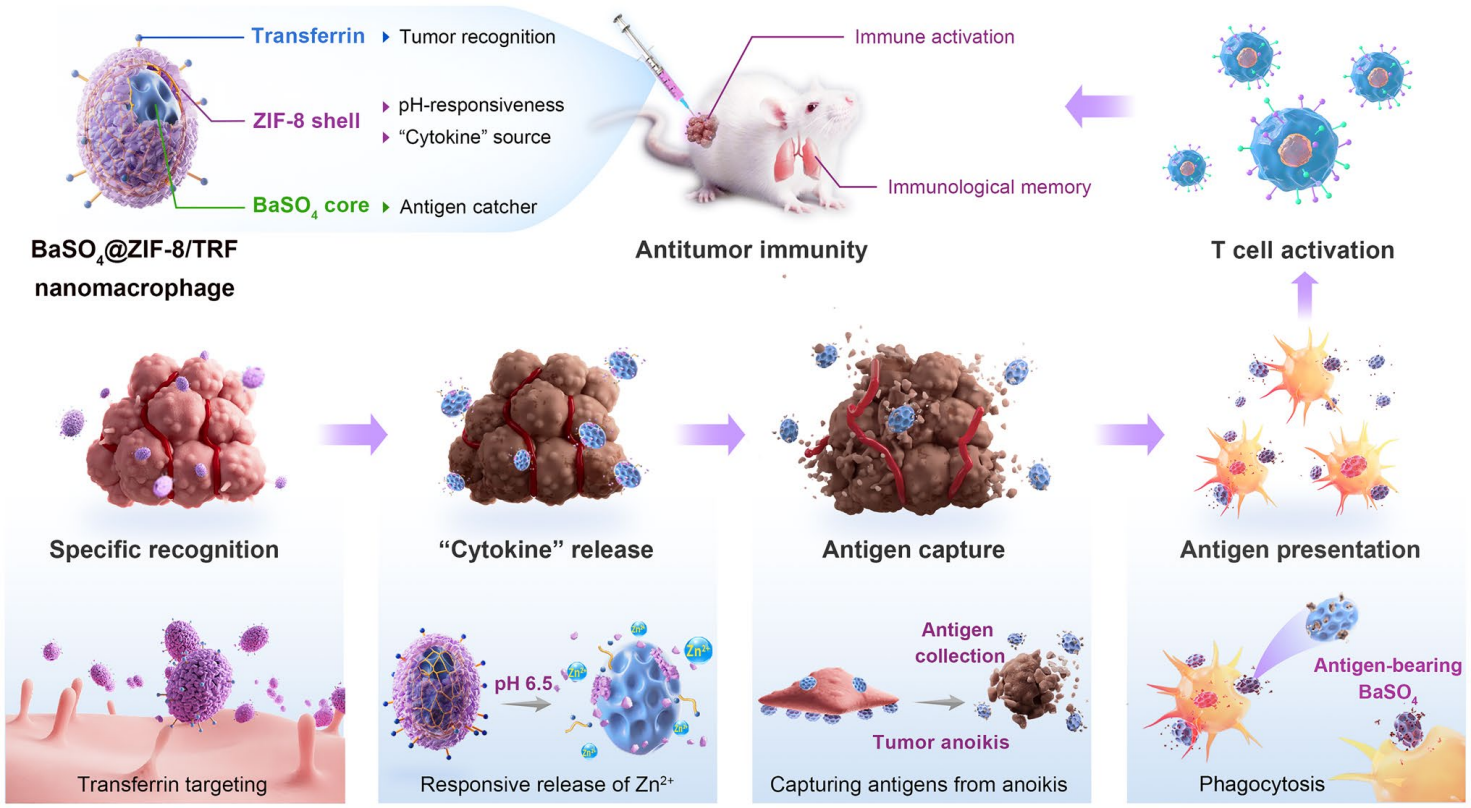
Schematic illustration of BaSO_4_@ZIF-8/TRF NMΦ for the modulation of antitumor immunity

Herein, a BaSO_4_@ZIF-8/TRF nanomacrophage (NMΦ) with hierarchical nanostructure is constructed as an alternative to immunoactive macrophages (Fig. 1). Upon residence in tumor via transferrin (TRF), the BaSO_4_@ZIF-8/TRF NMΦ can release Zn^2+^ as an “artificial cytokine” to reverse tumor immunosuppression and induce tumor anoikis. The released tumor antigens can be selectively captured by the cavities of BaSO_4_ nanoparticles, which is similar to the antigen capturing process of immunoactive macrophages. Thus, the BaSO_4_@ZIF-8/TRF NMΦ successfully mimics the basic functions of immunoactive macrophage, including tumor residence, cytokine release, antigen capture and immune activation. As a result, the NMΦ efficiently mediates macrophage polarization and T cell activation to fabricate systemic antitumor immunity. Besides, the immune memory established by the NMΦ can work against tumor recurrence.

## Experimental Section

### Materials

Polyacrylic acid (PAA), barium hydroxide octahydrate (Ba(OH)_2_•8H_2_O), sodium sulfide nonahydrate (Na_2_S·9H_2_O), zinc nitrate hexahydrate (Zn(NO_3_)_2_·6H_2_O), 2-methylimidazole, and isopropanol were obtained from Aladdin (Shanghai, China). The transferrin (TRF) was purchased from Yuanye Bio-Technology Co., Ltd (Shanghai, China). Dulbecco’s modified Eagle medium (DMEM), fetal bovine serum, 1% streptomycin/penicillin, phosphate buffered saline (PBS), and lipopolysaccharide (LPS) were obtained from Thermo Fisher (Beijing, China). The membrane protein extraction kit was purchased with Invent Biotechnologies (Eden Prairie, USA). FITC-/Cy3.5-/Cy5.5-fluorescence protein label kits, Cy3.5-PAA and Cy5.5-PAA were obtained from Ruixi Biological Technology Co., Ltd (Xi′an, China). BCA protein assay kit, 4% paraformaldehyde, phalloidin-iFluor 488, lactic dehydrogenase (LDH) cytotoxicity assay kit, radioimmunoprecipitation (RIPA) buffer lysis, phenylmethanesulfonyl fluoride (PMSF), HRP-labeled goat anti-rabbit IgG, Alexa Fluor 488-labeled goat anti-rabbit IgG, bovine serum albumin (BSA), D-luciferin, electrochemiluminescence (ECL) assay kit, cell counting kit-8 (CCK-8), calcein-AM, propidium iodide, reactive oxygen species assay kit, mitochondrial membrane potential assay kit, and cell cycle analysis kit were purchased from Beyotime (Haimen, China). Rabbit anti-E-cadherin, anti-integrin β1, anti-JNK, anti-BIM, anti-BMF, anti-BAK, anti-caspase 3, anti-Bcl-2, anti-Bax, anti-GAPDH antibodies were supplied by ABclonal Technology Co.,Ltd. (Wuhan, China). Chloroquine (CQ) was obtained from Sigma-Aldrich (St. Louis, MO, USA). CellTracker™ Green CMFDA, CellMaker™ Blue, Zinquin and 4′,6-diamidino-2′-phenylindole (DAPI) were obtained from Maokang Biotechnology Co.,Ltd. (Shanghai, China). Enzyme linked immunosorbent assay (ELISA) kits for TNF-α, IL-6, IL-10, ATP and IFN-γ were supplied by DAKEWE (Beijing, China). Anti-calreticulin (CRT), and anti-HMGB1 antibodies were purchased from Abcam (Cambridge, UK). FITC-conjugated CD86, APC-conjugated CD206, PE-conjugated CD80, APC-conjugated CD11c, PE-conjugated CD4, APC-conjugated CD8a, FITC-conjugated CD3, PE-conjugated F4/80, FITC-conjugated CD44, PerCP/Cy5.5-conjugated CD62L, and PE-conjugated CD8a antibodies were obtained from Biolegend (San Diego, USA). Anti-mouse PD-1 was supplied by Bioxcell (West Lebanon, USA).

### Synthesis of BaSO_4_ Nanoparticles

The PAA modified barium sulfate (BaSO_4_) nanoparticles were synthesized by chemical coprecipitation method. Briefly, 3.6 mg of PAA was added into 50 mL of Ba(OH)_2_·8H_2_O aqueous solution (1 mg mL^−1^) and vigorously stirred for 30 min. Then, PAA-Ba was gradually precipitated from the solution and collected by centrifugation (8000 g, 10 min). Subsequently, PAA-Ba was dispersed in 50 mL of water and mixed with 10 mL of Na_2_S·9H_2_O aqueous solution (20 mg mL^−1^). After reaction for 7 h at room temperature, the reaction solution was mixed with 15 mL of isopropanol and centrifugated (8000 g, 10 min) to obtain PAA-BaSO_4_ nanoparticles. Cy3.5-PAA and Cy5.5-PAA were used to synthesize fluorescense-labeled PAA-BaSO_4_ nanoparticles.

### Preparation and Modification of BaSO_4_@ZIF-8 Nanoparticles

Twenty milligrams of PAA-BaSO_4_ nanoparticles were dispersed in 30 mL of isopropanol, followed by the addition of 14.8 mg of Zn(NO_3_)_2_·6H_2_O. After stirring for 30 min, 10 mL of 2-methylimidazole (80 mM) in isopropanol was added and stirred for another 2 h. The BaSO_4_@ZIF-8 nanoparticles were collected by centrifugation (8000 g, 10 min). For modification of BaSO_4_@ZIF-8 with TRF, 10 mg of TRF was added into 20 mL of BaSO_4_@ZIF-8 nanoparticles in water (1 mg mL^−1^) and stirred overnight. The BaSO_4_@ZIF-8/TRF nanoparticles were collected by centrifugation (8000 g, 10 min). Similarly, the Cy3.5 or Cy5.5-labeled BaSO_4_@ZIF-8/TRF nanoparticles were also synthesized following the above mentioned procedures. To prepare ZIF-8/TRF nanoparticles, 14.8 mg of Zn(NO_3_)_2_·6H_2_O was dissolved in 30 mL of isopropanol, and then mixed with 10 mL of 2-methylimidazole (80 mM) in isopropanol for 30 min. ZIF-8 nanoparticles were collected by centrifugation (8000 g, 10 min) and modified with TRF following the above mentioned procedures.

### Characterizations

The morphology of nanomaterials was captured by transmission electron microscopy (TEM; JEM-2100F, JEOL, Japan) and scanning electron microscopy (SEM; NanoSEM430, FEI, Netherlands). Elemental compositions were determined on an X-Ray photoelectron spectroscopy (ESCALAB 250, Thermo, USA). The hydrodynamic size and zeta potential of nanomaterials were measured on a zeta nanosizer instrument (NanoBrook 90Plus, Brookhaven, USA). Crystal structures of nanomaterials were analyzed on a powder X-ray diffractometer (XRD) (X'pert Pro MPD, Philips, Netherlands). Fourier transform infrared (FT-IR) spectra were detected on an FT-IR spectrometer (Nicolet iS50, Thermo Fisher, USA). The absorption spectra were measured on a UV–Vis spectrometer (UV-2450, Shimadzu, Japan). The pore size was characterized with an automatic BET specific surface area and porosity analyzer (ASAP2020 HD88 type, Micromeritics, USA).

### Cell Culture and Animal Model

All the cells were cultured in complete DMEM medium with 10% fetal bovine serum and 1% streptomycin/penicillin at 37 °C in a 5% CO_2_ atmosphere. Animal experiments were carried out under protocols (No. XMSB-2022–0103) approved by the Department of Laboratory Animals (Central South University).

### Behaviors of Nanomaterials in Varied pH

BaSO_4_, ZIF-8/TRF, BaSO_4_@ZIF-8 and BaSO_4_@ZIF-8/TRF nanomacrophages (NMΦs) were dispersed in 20 mL of PBS buffer with different pH (7.4 and 6.5) at 37 °C. At desired time points (0, 2, 4, 8, 12 and 24 h), 1 mL of solution in different groups was taken out and centrifuged at 8000 g for 10 min. The contents of Zn^2+^ and Ba^2+^ in the supernatants were detected by an atomic absorption spectrometer (TAS-990AFG, Puxi, China).

The 4T1 cells were treated with BaSO_4_ (120 μg mL^−1^), ZIF-8/TRF (30 μg mL^−1^), BaSO_4_@ZIF-8 (150 μg mL^−1^), and BaSO_4_@ZIF-8/TRF (150 μg mL^−1^) for 18 h. Membrane proteins of 4T1 tumor cells were extracted with membrane protein extraction kit following the manufacturer’s protocol. To study the protein trapping capacity and binding selectivity of different nanomaterials, TRF, BSA and membrane proteins were individually labeled with FITC, Cy3.5 and Cy5.5 using fluorescence label kits. Then, 10 mL of BaSO_4_, ZIF-8/TRF-FITC, BaSO_4_@ZIF-8 and BaSO_4_@ZIF-8/TRF-FITC nanoparticles (1 mg mL^−1^) were mixed with 5 mL of Cy3.5-BSA (1 mg mL^−1^) or Cy5.5-membrane proteins (1 mg mL^−1^) in phosphate buffer (pH = 7.4 or 6.5). After 24 h, the mixture solution was taken out and centrifugated at 10,000 g for 10 min. The protein contents in both the supernatants and precipitations were quantified with BCA protein assay kit. Then, the different protein contents in the supernatants and precipitations were quantified by fluorescence measurement with a microplate reader (Infinite E Plex, Tecan, Switzerland).

### Tumor Cell Anoikis

The 4T1 cells and L02 cells were treated with BaSO_4_ (120 μg mL^−1^), ZIF-8/TRF (30 μg mL^−1^), BaSO_4_@ZIF-8 (150 μg mL^−1^), and BaSO_4_@ZIF-8/TRF (150 μg mL^−1^) for 18 h. Then, cells were fixed in 4% paraformaldehyde and observed with SEM. To further study the cytoskeleton structure, 4T1 cells were seeded into a 6-well plate and treated with BaSO_4_ (120 μg mL^−1^), ZIF-8/TRF (30 μg mL^−1^), BaSO_4_@ZIF-8 (150 μg mL^−1^), BaSO_4_@ZIF-8/TRF (150 μg mL^−1^), and BaSO_4_@ZIF-8/TRF (150 μg mL^−1^) + CQ (3 μg mL^−1^) for 12 h. The stress fibers were stained with phalloidin-iFluor 488 following the manufacturer’s protocol. The fluorescence images were captured with an inverted fluorescence microscope (IX83, Olympus, Japan).

Western blot assay was performed to detect the variations of anoikis-related proteins. The 4T1 cells were cultured in a 6-well plate and incubated with BaSO_4_ (120 μg mL^−1^), ZIF-8/TRF (30 μg mL^−1^), BaSO_4_@ZIF-8 (150 μg mL^−1^), and BaSO_4_@ZIF-8/TRF (150 μg mL^−1^), and BaSO_4_@ZIF-8/TRF (150 μg mL^−1^) + CQ (3 μg mL^−1^) for 24 h. The cell supernatants were collected for cell viability detection using LDH cytotoxicity assay kit. Then, all the cells were collected and lysed with RIPA lysis buffer (containing 1 mM PMSF) on the ice for 30 min. After centrifugation (12,000 g, 4 °C) for 20 min, the obtained supernatants were stored at − 80 °C. Membrane proteins were extracted with membrane protein extraction kit according to the manufacturer’s protocols. The proteins were resolved by the sodium dodecyl sulfate–polyacrylamide gel electrophoresis (SDS-PAGE), and then transferred onto the surface of polyvinylidene difluoride (PVDF) membranes. Subsequently, PVDF membranes were blocked with 5% BSA and co-incubated with different rabbit anti-mouse primary antibodies (*i.e.,* anti-E-cadherin, anti-integrin β1, anti-JNK, anti-BIM, anti-BMF, anti-BAK, anti-caspase 3, anti-Bcl-2, anti-Bax, and anti-GAPDH) at 4 °C overnight. After washing with TBST three times, the membranes were treated with HRP-labeled goat anti-rabbit IgG at room temperature for 1 h. The protein bands were imaged with a chemiluminescence gel imaging system (Universal Hood II, BIO-RAD, USA) using an electrochemiluminescence (ECL) assay kit.

### Cell Viability

4T1, 3T3 and L02 cells were incubated in 96-well plates and treated with BaSO_4_ (30, 60, 90, 120 μg mL^−1^), ZIF-8/TRF (10, 20, 30, 40 μg mL^−1^), BaSO_4_@ZIF-8 (60, 90, 120, 150 μg mL^−1^), BaSO_4_@ZIF-8/TRF (60, 90, 120, 150 μg mL^−1^), and BaSO_4_@ZIF-8/TRF (60, 90, 120, 150 μg mL^−1^) + CQ (3 μg mL^−1^) for 24 h. Cytotoxicity was detected with CCK-8 assay kit following the manufacturer’s protocol. Cells were stained with calcein-AM (10 μM) and propidium iodide (20 μM) for 20 min. Fluorescent images were captured with the inverted fluorescence microscope.

To study the intracellular Zn^2+^ content, 4T1, 3T3 and L02 cells were treated with BaSO_4_ (120 μg mL^−1^), ZIF-8/TRF (30 μg mL^−1^), BaSO_4_@ZIF-8 (150 μg mL^−1^), BaSO_4_@ZIF-8/TRF (150 μg mL^−1^), and BaSO_4_@ZIF-8/TRF (150 μg mL^−1^) + CQ (3 μg mL^−1^) for different time periods (4, 8 and 12 h). Then, all the cells were collected and stained with Zinquin (20 μM) for 30 min. The intracellular fluorescence was analyzed on a flow cytometer (Novocyte 2040R, Agilent, USA).

### Intracellular Oxidative Stress and Cell Cycle Analysis

4T1 cells were treated with BaSO_4_ (120 μg mL^−1^), ZIF-8/TRF (30 μg mL^−1^), BaSO_4_@ZIF-8 (150 μg mL^−1^), BaSO_4_@ZIF-8/TRF (150 μg mL^−1^), and BaSO_4_@ZIF-8/TRF (150 μg mL^−1^) + CQ (3 μg mL^−1^) for 12 h. To detect mitochondrial membrane potential, cells were stained with JC-1 assay kit following the manufacturer’s protocol. To detect intracellular oxidative stress, cells were stained with DCFH-DA (10 μM) for 20 min. Cellular fluorescence was imaged with an inverted fluorescence microscope.

4T1 cells were treated with BaSO_4_ (120 μg mL^−1^), ZIF-8/TRF (30 μg mL^−1^), BaSO_4_@ZIF-8 (150 μg mL^−1^), BaSO_4_@ZIF-8/TRF (150 μg mL^−1^), and BaSO_4_@ZIF-8/TRF (150 μg mL^−1^) + CQ (3 μg mL^−1^) for 24 h. After fixation with 70% ethanol overnight, cell cycles were analyzed by the flow cytometer with a cell cycle analysis kit.

### Immunogenic Cell Death

4T1 cells were cultured in a 48-well confocal plate and treated with BaSO_4_ (120 μg mL^−1^), ZIF-8/TRF (30 μg mL^−1^), BaSO_4_@ZIF-8 (150 μg mL^−1^), BaSO_4_@ZIF-8/TRF (150 μg mL^−1^), and BaSO_4_@ZIF-8/TRF (150 μg mL^−1^) + CQ (3 μg mL^−1^) for 12 h. The supernatant in each well was collected for ATP quantification using an ATP assay kit. Moreover, 4T1 tumor cells were fixed with 4% paraformaldehyde, and permeabilized with 0.5% Triton-X100. Cells were immunostained with rabbit anti-mouse CRT or HMGB1 antibodies at 4 °C overnight. After washing with PBS three times, cells were stained with Alexa Fluor 488-labeled goat anti-rabbit IgG for another 1 h at room temperature. Finally, the nuclei were stained with DPAI (1 μM) for 30 min, and the fluorescent images were taken with a high-content cell imaging system (Operetta, Platinum Elmer Instruments, USA).

### Macrophage Polarization

4T1 tumor cells and J774A.1 macrophages were individually stained with CellTracker™ Green CMFDA and CellMaker™ Blue CMAC following the manufacturer’s protocols. Green fluorescence-labeled 4T1 cells and blue fluorescence-labeled J774A.1 macrophages were seeded into the upper and lower chambers of transwells, respectively. After incubation overnight, cell culture medium in the upper chambers was replaced by medium containing Cy3.5-BaSO_4_ (120 μg mL^−1^), ZIF-8/TRF (30 μg mL^−1^), Cy3.5-BaSO_4_@ZIF-8 (150 μg mL^−1^), and Cy3.5-BaSO_4_@ZIF-8/TRF (150 μg mL^−1^). After 24 h of incubation, the fluorescence co-localization images were captured by a high-content cell imaging system.

For macrophage polarization study, 4T1 cells in the upper chambers were treated with LPS (1 μg mL^−1^), BaSO_4_ (120 μg mL^−1^), ZIF-8/TRF (30 μg mL^−1^), BaSO_4_@ZIF-8 (150 μg mL^−1^), and BaSO_4_@ZIF-8/TRF (150 μg mL^−1^) for 24 h, respectively (n = 5 in each group). The cytokines (including IL-10, TNF-α and IL-6) in the supernatants of macrophages were detected with corresponding ELISA assay kits. The J774A.1 macrophages in the lower chambers were stained with FITC-conjugated CD86 and APC-conjugated CD206 antibodies. The polarization of M1 (CD86^high^/CD206^low^) and M2 (CD86^low^/CD206^high^) phenotypes was detected by flow cytometry.

### In vivo Immune Responses

Female BALB/c mice (6-week-old) were subcutaneously injected with 4T1 tumor cells to fabricate bilateral tumors model. When the primary tumor volume was over 50 mm^3^, mice received different treatments (n = 5 in each group): (1) PBS, (2) BaSO_4_, (3) ZIF-8/TRF, (4) BaSO_4_@ZIF-8, (5) BaSO_4_@ZIF-8/TRF. All the nanoparticles were intratumorally injected into the primary tumors at the dosage of 5 mg kg^−1^. On day 3 post injection, mouse blood was collected and the serum cytokines (TNF-α, IL-6, IL-10, and INF-γ) were detected with corresponding ELISA assay kits. On day 5 post injection, Zinquin was injected into the primary tumors at the dosage of 2 mg kg^−1^ for Zn^2+^ detection. Moreover, the spleens and primary tumors were isolated to prepare single-cell suspensions. All the cells were fixed with 4% paraformaldehyde and blocked in 5% bovine serum albumin (BSA). To detect dendritic cell maturation, the splenic cell suspensions were stained with FITC-conjugated CD86, PE-conjugated CD80, and APC-conjugated CD11c at 4 °C for 12 h. To detect T cell activation, the splenic cell suspensions were stained with PE-conjugated CD4, APC-conjugated CD8a, and FITC-conjugated CD3 at 4 °C for 12 h. To detect the intratumoral macrophages, cell suspensions of primary tumors were stained with FITC-conjugated CD86, APC-conjugated CD206 and PE-conjugated F4/80 at 4 °C for 12 h. All the cell suspensions were washed with PBS three times and analyzed by flow cytometry. In addition, the distant tumors were sliced and subjected to CD3/CD4/CD8 immunohistological staining and FoxP3/CD3 immunofluorescent staining.

### Antitumor Therapy in vivo

4T1-Luc tumor cells were subcutaneously injected into mice to fabricate bilateral tumor model. When the primary tumor volume was over 50 mm^3^, mice were divided into eight groups (n = 6 in each group): (1) PBS; (2) BaSO_4_; (3) anti-PD-1; (4) ZIF-8/TRF; (5) BaSO_4_@ZIF-8; (6) BaSO_4_@ZIF-8/TRF; (7) ZIF-8/TRF + anti-PD-1; (8) BaSO_4_@ZIF-8/TRF + anti-PD-1. All the nanoparticles were intratumorally injected into the primary tumors at the dosage of 5 mg kg^−1^. Anti-PD-1 was intravenously injected into mice at the dosage of 0.75 mg kg^−1^ on day 1, 4, and 7. To monitor tumor growth, tumor bioluminescence images were captured after intravenous injection of D-luciferin (100 mg kg^−1^) every 5 days by an IVIS Lumina imaging system (PerkinElmer, USA). The tumor volumes of mice were recorded by a vernier caliper every other day and calculated according to the following formula: tumor volume = (width^2^ × length)/2; meanwhile the body weights of mice were measured. On day 15 post injection, blood cells were harvested for blood biochemistry analysis on an auto hematology analyzer (HF-3800, HLIFE, China) and a blood chemistry analyzer (Pointcare V2, MNCHIP, China). All tumors were subjected to H&E and TUNEL staining for histopathological analysis. Main organs (liver, heart, spleen, lung, kidney) were also excised and stained with H&E. To calculate survival rate, mice received the above treatments and were fed for 60 days. When tumor volume was over 2000 mm^3^, mice were euthanized according to ethical guidelines. The survival rates of mice were calculated on day 60 post-injection.

For tumor anoikis analysis, the harvested primary tumors in different groups were divided into two halves. One half of the tumors were fixed and sliced for immunofluorescence staining of E-cadherin and integrin β1. The other half was prepared into single-cell suspension for western blotting analysis following the aboved-mentioned procedures.

### Immune Memory Effect

4T1 breast tumor-bearing mice received the above-mentioned treatments for 15 days (n = 10 in each group). Residual tumors were removed via surgical excision under anesthesia. On day 28 post-treatment, mice were intravenously injected with 4T1-Luc cells (1 × 10^6^). On day 32, the spleens of one half of the mice were harvested to obtain single-cell suspensions. Following tissue homogenization and erythrocyte lysis, the cells were stained with FITC-conjugated CD44, PerCP/Cy5.5-conjugated CD62L and PE-conjugated CD8a. On day 42 post-treatment, D-luciferin (100 mg kg^−1^) was intravenously injected into the mice, and the bio-luminescence images were captured by the IVIS Lumina imaging system. Finally, the lungs of mice were collected and the tumor metastases were histologically examined via H&E staining.

### Biodistributions in vivo

4T1 tumor cells were subcutaneously injected into mice to fabricate unilateral tumor model. 4T1 breast tumor-bearing mice received intratumoral injection of Cy5.5-BaSO_4_@ZIF-8 or Cy5.5-BaSO_4_@ZIF-8/TRF at a dose of 5 mg kg^−1^. The nanoparticles in vivo were tracked through fluorescence imaging on the IVIS Lumina imaging system. At 24 h post injection, mice were euthanized for organ collection. The biodistributions of nanoparticles in the main organs were detected on the IVIS Lumina imaging system. For pharmacokinetics study of BaSO_4_@ZIF-8 nanoparticles, Cy5.5-BaSO_4_@ZIF-8 and Cy5.5-BaSO_4_@ZIF-8/TRF were intravenously injected into mice at a dose of 5 mg kg^−1^. The nanoparticles in vivo were tracked through fluorescence imaging. At 72 h post injection, the main organs of mice were harvested for the Ba content analysis.

### Statistical Analysis

All the data were expressed as the mean ± standard deviation (SD). The experiments were replicated three times, unless otherwise stated. The normality of data was examined by Shapiro–Wilk tests in SPSS Statistics 26. One-way ANOVA of variance was utilized to evaluate significance of difference. * *p* < 0.05, and ** *p* < 0.01.

## Results and Discussion

### Preparation and Characterization

**Fig. 2:**
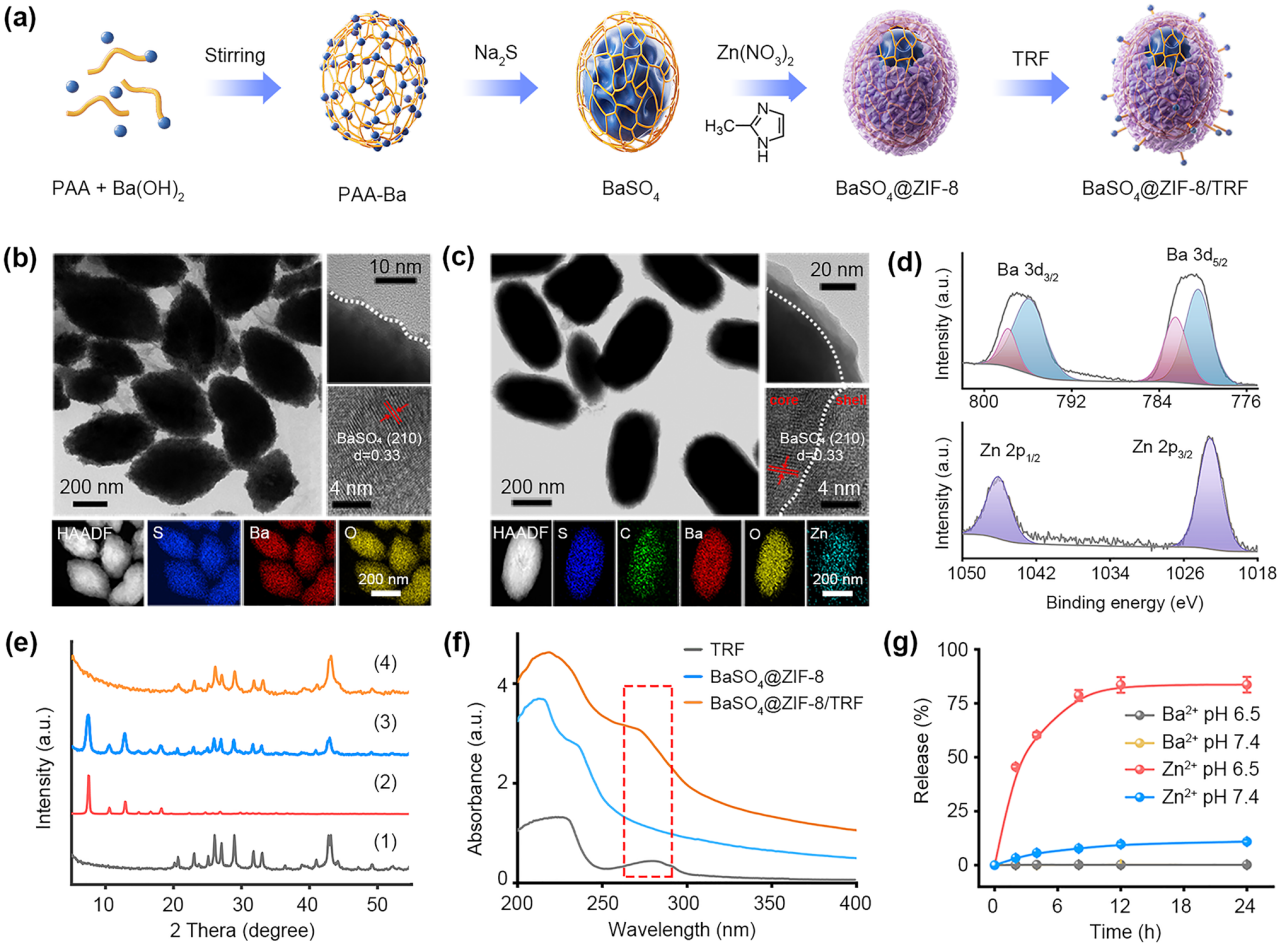
Characterization of BaSO_4_@ZIF-8/TRF NMΦ. **a** Schematic diagram of the preparation of BaSO_4_@ZIF-8/TRF NMΦ. **b** TEM images and elemental mappings of BaSO_4_, and **c** BaSO_4_@ZIF-8 nanoparticles. **d** XPS spectra of BaSO_4_@ZIF-8 nanoparticles. **e** XRD patterns of (1) BaSO_4_, (2) ZIF-8, (3) BaSO_4_@ZIF-8 (pH 7.4), and (4) BaSO_4_@ZIF-8 (pH 6.5). **f** UV–Vis spectra of TRF, BaSO_4_@ZIF-8 nanoparticles, and BaSO_4_@ZIF-8/TRF NMΦs. **g** pH-responsive release of Ba^2+^ and Zn^2+^ from BaSO_4_@ZIF-8/TRF NMΦs

To assemble BaSO_4_@ZIF-8 hierarchical structures, BaSO_4_ nanoparticles were initially prepared via a chemical precipitation method (Fig. 2a). As displayed in transmission electron microscopy (TEM) results, BaSO_4_ nanoparticles are uniformly dispersed nanoellipsoids with average size of 323 ± 5 nm in length and aspect ratio of 2.2:1 (Fig. 2b). Lattice fringes with a spacing of 0.33 nm can be detected, corresponding to the (210) plane of BaSO_4_. Elemental mapping results reveal the overlapping distribution of Ba, S and O elements in the BaSO_4_ nanoparticles. The obtained BaSO_4_ nanoparticles are coated with poly acrylic acid (PAA) at the surface (Fig. S1), which can serve as the template for ZIF-8 growth. After the in situ growth of ZIF-8, an obvious shell with thickness of 8 nm can be clearly observed at the surface of BaSO_4_ nanoparticles (Fig. 2c). The elemental mapping images verify the presence of Zn element on the surface of nanocomposites, suggesting the successful preparation of BaSO_4_@ZIF-8 nanoparticles. Similar morphology and element distributions are reflected by SEM images (Fig. S2). X-ray photoelectron spectroscopy (XPS) data reveal the coexistence of Ba and Zn with atomic ratio of 1.6:1 in the BaSO_4_@ZIF-8 nanoparticles (Figs. 2d & S3). For comparison, ZIF-8 nanoparticles were synthesized according to the previous reports (Fig. S4) [30]. The powder X-ray diffraction (PXRD) analysis reveals that both the diffraction peaks of BaSO_4_ and ZIF-8 coexist in BaSO_4_@ZIF-8 nanoparticles (Figs. 2e & S5). Moreover, BaSO_4_@ZIF-8 nanoparticles can be dispersed well in the physiological solution for more than 7 days (Fig. S6).

Natural macrophages are usually distributed around tumor cells with high tumor-resident ability [31]. To realize tumor residence of BaSO_4_@ZIF-8 nanoparticles, TRF with specific affinity to tumor cells was decorated at the surface[32]. After the surface functionalization with positively charged TRF, the zeta potential of BaSO_4_@ZIF-8/TRF is − 22.4 ± 0.9 mV, which is higher than that of BaSO_4_@ZIF-8 nanoparticles (− 29.8 ± 0.6 mV) (Table S1). Besides, the UV–vis absorption spectrum of BaSO_4_@ZIF-8/TRF displays an obvious absorption band of TRF at 272 nm (Fig. 2f), validating the successful decoration of TRF. The quantitative result suggests that approximately 78.8 mg g^−1^ of TRF is decorated on the BaSO_4_@ZIF-8 nanoparticles. BaSO_4_@ZIF-8/TRF NMΦs also possess stable dispersion within 7 days in the physiological solution (Fig. S7).

### Artificial Cytokine Role of Zn^2+^

**Fig. 3:**
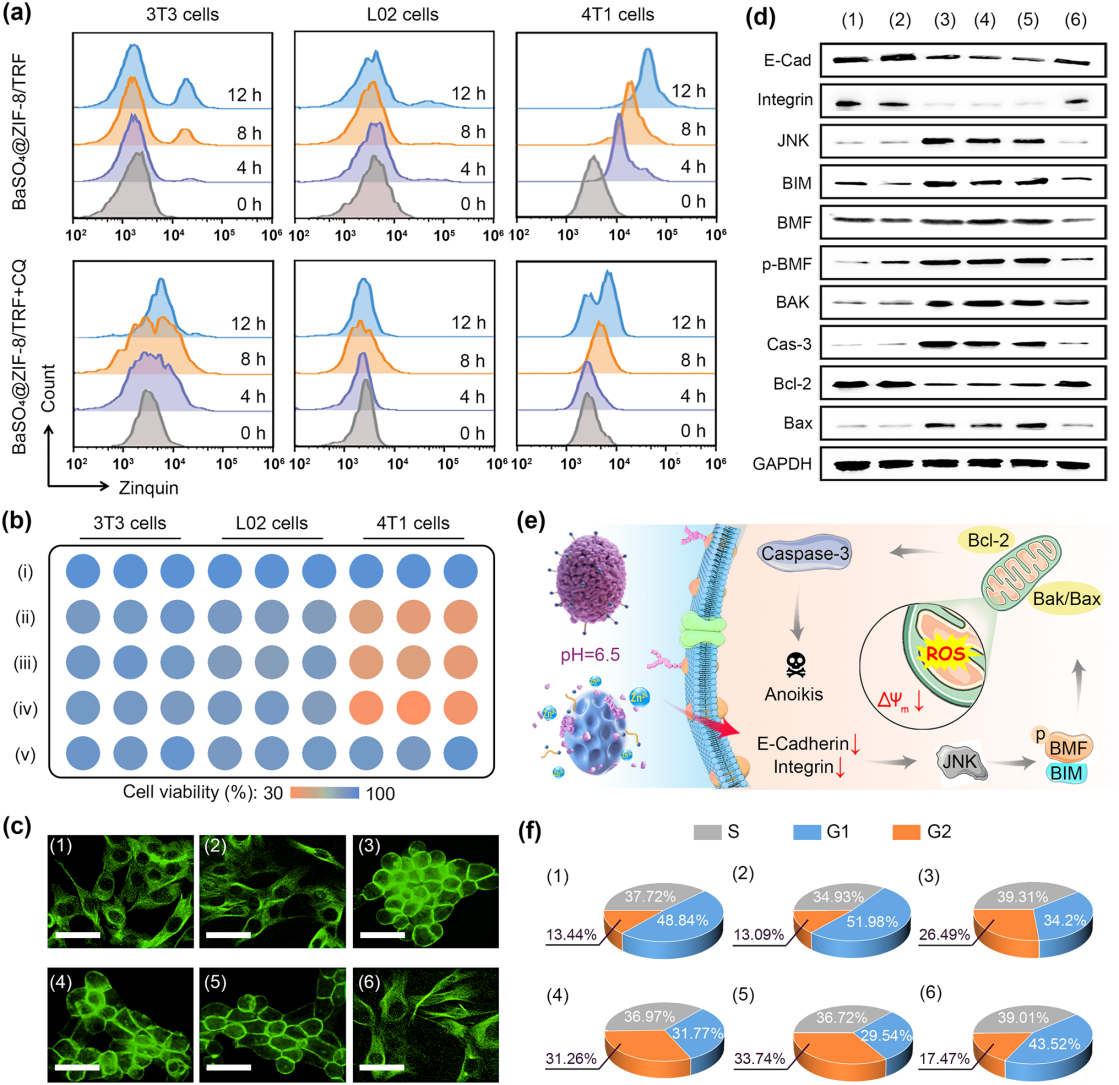
Artificial cytokine release and tumor cell anoikis. **a** Intracellular Zn^2+^ content of 4T1, 3T3 and L02 cells after incubation with BaSO_4_@ZIF-8/TRF and BaSO_4_@ZIF-8/TRF + CQ for different time periods. **b** Cytotoxicity of BaSO_4_@ZIF-8/TRF NMΦs to both normal cells and tumor cells: (i) blank control, (ii) BaSO_4_@ZIF-8/TRF (60 μg mL^−1^), (iii) BaSO_4_@ZIF-8/TRF (90 μg mL^−1^), (iv) BaSO_4_@ZIF-8/TRF (150 μg mL^−1^), (v) BaSO_4_@ZIF-8/TRF (150 μg mL^−1^) + CQ (3 μg mL^−1^). **c** The fluorescence images of cytoskeletons of tumor cells after different treatments (scale bars = 20 μm). **d** Western blotting analysis of cellular expressions of anoikis-related proteins in 4T1 cells after different treatments. **e** Schematic illustration of the anoikis mechanism of tumor cells induced by BaSO_4_@ZIF-8/TRF NMΦs. **f** Cell cycle distributions of tumor cells after different treatments. Group: (1) control; (2) BaSO_4_; (3) ZIF-8/TRF; (4) BaSO_4_@ZIF-8; (5) BaSO_4_@ZIF-8/TRF; (6) BaSO_4_@ZIF-8/TRF + CQ

The immune response of macrophages highly relies on cytokine secretion to regulate tumor cell death and immune cell activation [33]. To verify the possibility of Zn^2+^ as an artificial proinflammatory cytokine, we investigated Zn^2+^ release from the NMΦs in response to tumor pathological conditions, and its regulation on tumor cell death and immune activation. The PXRD data shows that the diffraction peaks of ZIF-8 disappear when BaSO_4_@ZIF-8 nanoparticles are exposed to tumor acidic condition (pH 6.5) (Fig. 2e). Due to the acid-responsive decomposition of ZIF-8, 83.7% of Zn^2+^ ions can be released from BaSO_4_@ZIF-8/TRF at tumor acidic condition. By contrast, the Zn^2+^ release was barely detected under normal physiological condition (pH 7.4) (Fig. 2g). To investigate Zn^2+^ release in vitro, BaSO_4_@ZIF-8/TRF NMΦs were co-incubated with tumor cells and normal cells, respectively. Similarly, the cumulative release rates of Zn^2+^ from BaSO_4_@ZIF-8/TRF NMΦs under tumor acidic and normal physiological conditions were 67% and 16%, respectively. Comparatively, Ba^2+^ release was negligible either at tumor acidic conditions or at normal physiological conditions, indicating the high stability of BaSO_4_ (Fig. S8). The intracellular Zn^2+^ flux was further investigated in tumor cells (4T1), and normal cells (3T3 and L02) in vitro. The cellular Zn^2+^ was stained with Zinquin as a fluorescent probe and quantified with flow cytometry (Figs. 3a & S9). When 4T1 tumor cells were treated with BaSO_4_@ZIF-8/TRF for 12 h, the cellular Zinquin fluorescence was approximately increased by 10 times, which is much higher than those of 3T3 and L02 cells. To confirm Zn^2+^ accumulation in tumor cells, chloroquine (CQ) as a typical Zn^2+^ inhibitor was supplemented to scavenge free Zn^2+^. Obviously, the intracellular Zinquin fluorescence of tumor cells returned to normal level. Thus, BaSO_4_@ZIF-8/TRF NMΦ can release Zn^2+^ ions under tumor acidic conditions, similar to the cytokines secreting profiles of macrophages.

Proinflammatory cytokines are able to mediate tumor cell death via multiple apoptotic pathways [34–36]. To determine the tumor-killing activity of Zn^2+^ as an artificial proinflammatory cytokine, cell viability assay was carried out on 4T1, 3T3 and L02 cells. After the treatment of BaSO_4_@ZIF-8/TRF NMΦs, the cell viabilities of 3T3 and L02 cells maintained above 80% (Figs. 3b & S10); while more than 70% of 4T1 tumor cells were killed after the same treatments. The tumor-killing activity of BaSO_4_@ZIF-8/TRF NMΦs was also validated by the live-dead staining (Fig. S11). Obviously, the majority of tumor cells were dead after the treatment of BaSO_4_@ZIF-8/TRF. Notably, the toxicity was also observed in the groups of ZIF-8/TRF and BaSO_4_@ZIF-8 but not in the group of BaSO_4_ nanoparticles, indicating the Zn^2+^-mediated tumor cytotoxicity. To confirm this assumption, CQ was used as an inhibitor to scavenge free Zn^2+^. As expected, tumor cell viability was recovered to 91% in the co-presence of BaSO_4_@ZIF-8/TRF NMΦs and CQ, verifying the cytotoxic role of Zn^2+^. Therefore, Zn^2+^ can mimic the function of proinflammatory cytokines to mediate tumor cell death.

### BaSO_4_@ZIF-8/TRF NMΦ Mediated Tumor Anoikis

To understand the antitumor mechanism of BaSO_4_@ZIF-8/TRF NMΦ, cytoskeleton of tumor cells was examined with fluorescent phalloidin as a specific probe. As shown in Fig. 3c, cytoskeleton collapse and cell detachment of the tumor cells were clearly found after the treatment of BaSO_4_@ZIF-8/TRF NMΦs, which are the characteristics of anoikis [37]. To further confirm the anoikis of tumor cells, cellular adhesion-related proteins were examined through Western blotting. Evidently, as typical adhesion-related membrane proteins, E-cadherin and integrin β1 are individually down-regulated by 55% and 67% in the presence of BaSO_4_@ZIF-8/TRF NMΦs, respectively (Figs. 3d & S12). In general, cell adhesion was closely associated with cJUN NH2-terminal kinase (JNK) pathway [38]. In the presence of BaSO_4_@ZIF-8/TRF NMΦs, the reduced expression of adhesion-related proteins results in the 3.4-fold upregulation of JNK. As the downstream of JNK pathway, phosphorylated BMF and BIM are elevated by BaSO_4_@ZIF-8/TRF NMΦs, thereby activating BAK/BAX apoptotic pathway [39]. As a result, caspase-3 as an intracellular apoptosis effector is up-regulated 2.6-fold, while the expression of antiapoptotic Bcl-2 is suppressed by nearly 50% (Fig. S12). Based on the above evidence, tumor anoikis induced by BaSO_4_@ZIF-8/TRF NMΦs can be mediated by the JNK and BAK/BAX signaling pathways. It is noteworthy that the activation of JNK and BAK/BAX pathways was also observed in the groups of ZIF-8/TRF and BaSO_4_@ZIF-8, but not in the group of BaSO_4_, suggesting the pivotal role of Zn^2+^ in the processes. According to recent studies, cell adhesion is influenced by intracellular Ca^2+^ oscillations, which are highly activated in the presence of Zn^2+^ overload [40, 41]. Thus, tumor anoikis was deduced to be mediated by Zn^2+^ released from the decomposition of BaSO_4_@ZIF-8/TRF NMΦs. To validate the assumption, CQ as a Zn^2+^ scavenger was supplemented together with BaSO_4_@ZIF-8/TRF NMΦs. As expected, the expression of either E-cadherin or integrin β1 was recovered back to the normal level (Fig. S12); meanwhile, the activation of JNK and BAK/BAX pathways was suppressed. Therefore, the anoikis of tumor cells in the presence of BaSO_4_@ZIF-8/TRF NMΦs was mediated by Zn^2+^ release via activating JNK and BAK/BAX pathways (Fig. 3e).

Accumulating evidences indicate that anoikis is correlated with mitochondrial dysfunction [42]. Thus, mitochondrial membrane potential was examined by JC-1 probe, which tends to aggregate within intact mitochondria and emits red fluorescence. Upon mitochondria dysfunction, JC-1 would diffuse into cytoplasm and disassemble into monomer with green fluorescence. The red-to-green fluorescence ratio of tumor cells was remarkably decreased by 80% in the presence of BaSO_4_@ZIF-8/TRF NMΦs (Fig. S13), indicating mitochondrial dysfunction. Given the critical role of mitochondria in cellular redox homeostasis, mitochondrial dysfunction would result in cellular oxidative stress and lactic dehydrogenase (LDH) leakage [43]. The intracellular oxidative stress was detected with dichlorodihydrofluorescein diacetate (DCFH-DA) as a fluorescent probe. Obviously, bright green fluorescence was displayed in tumor cells after co-incubation of ZIF-8/TRF, BaSO_4_@ZIF-8 and BaSO_4_@ZIF-8/TRF; whereas little intracellular fluorescence was observed after the addition of BaSO_4_ or BaSO_4_@ZIF-8/TRF + CQ, suggesting Zn^2+^-induced intracellular oxidative stress (Fig. S14). Moreover, the LDH efflux increased almost 1.7-fold after the treatment of BaSO_4_@ZIF-8/TRF NMΦs (Fig. S15). Due to the mitochondrial damage, about 33.7% of tumor cells were retarded at the G2/M phase by BaSO_4_@ZIF-8/TRF NMΦs (Figs. 3f & S16). Notably, CQ as a Zn^2+^ chelator suppressed the mitochondrial dysfunction and cell cycle arrest induced by BaSO_4_@ZIF-8/TRF NMΦs. Thus, the Zn^2+^ released from BaSO_4_@ZIF-8/TRF NMΦs induced mitochondrial dysfunction and cell cycle arrest, giving rise to cell anoikis (Fig. 3e). Taken together, Zn^2+^ can be released from the NMΦs under tumor pathological condition and mediate tumor death via anoikis. All these features allow Zn^2+^ to be an artificial proinflammatory cytokine.

**Fig. 4:**
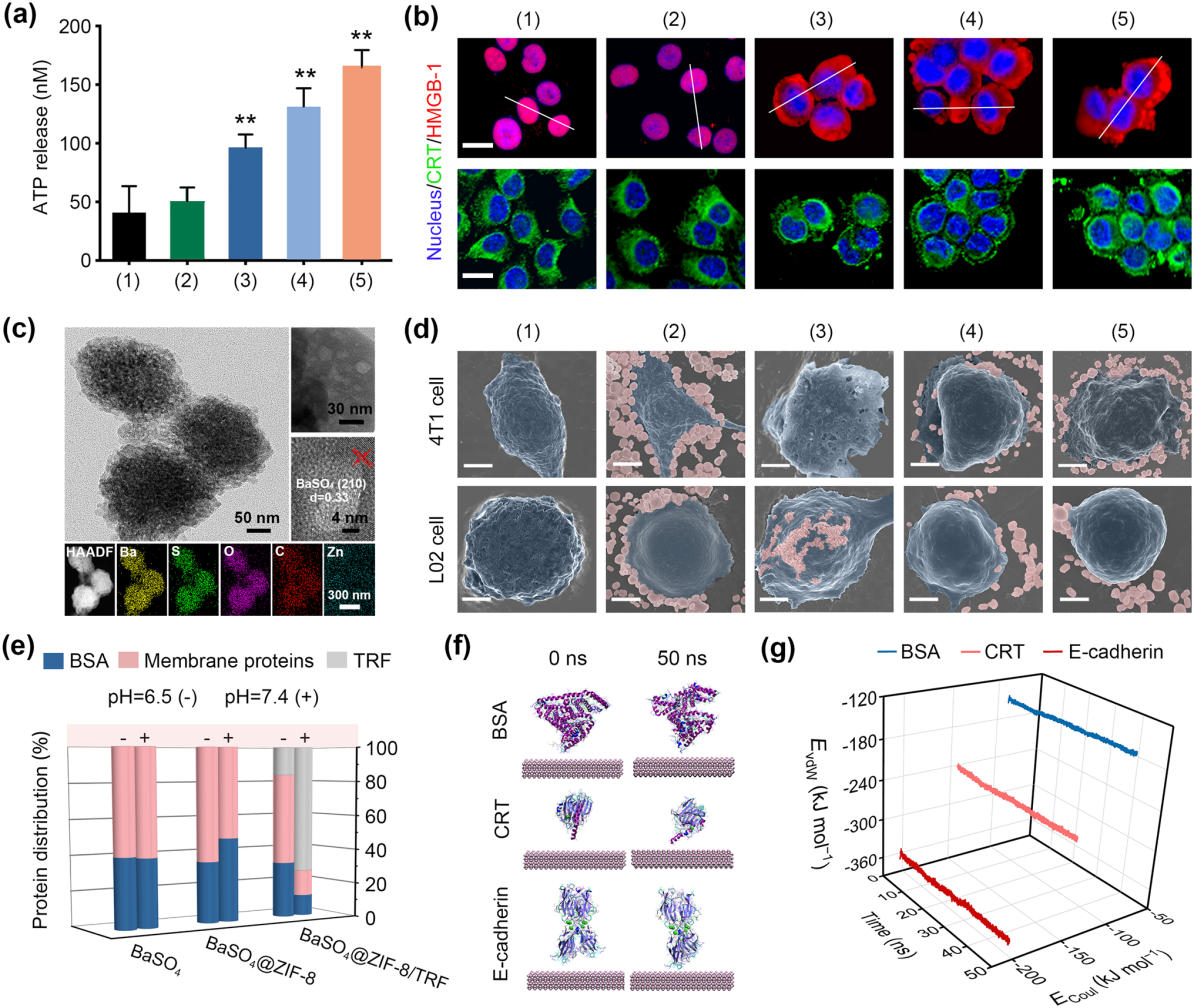
ICD effect and selective antigen capture of BaSO_4_@ZIF-8/TRF NMΦs. **a** ATP release from tumor cells after different treatments (n = 3). **p < 0.01 (compared to the control group). Groups: (1) control, (2) BaSO_4_, (3) ZIF-8/TRF, (4) BaSO_4_@ZIF-8, and (5) BaSO_4_@ZIF-8/TRF NMΦs. **b** Confocal microscopy images showing enrichment of CRT on the surface of cytoplasmic membrane, and translocation of HMGB1 in 4T1 tumor cells after different treatments. **c** TEM images and elemental mapping of BaSO_4_@ZIF-8/TRF at pH 6.5. **d** SEM pseudo color images of 4T1 cells and L02 cells after different treatments (blue-gray: cells; purple: nanoparticles, scale bars = 2 μm). **e** Binding capacity of different nanoparticles to BSA, membrane proteins and TRF. **f** MD simulations of the protein configurations on BaSO_4_ substrates within 50 ns. **g** Coulomb interaction energy (*E*_Coul_) and van der Waals energy (*E*_vdW_) of different proteins on BaSO_4_ substrates within 50 ns

The fabrication of adaptive immunity requires the immunogenic death of tumor cells to release damage associated molecular patterns (DAMPs), including adenosine triphosphate (ATP), calreticulin (CRT) and high-mobility group protein B1 (HMGB1) [44]. Natural macrophages can secrete proinflammatory cytokines such as tumor necrosis factor (TNF) to initiate immunogenic cell death of tumors [45]. Therefore, we examined the potential of Zn^2+^ as an artificial proinflammatory cytokine in triggering the release of DAMPs from tumor cells. Upon co-incubation with BaSO_4_@ZIF-8/TRF, the ATP release from 4T1 tumor cells gradually increased fourfold, as compared with the control group (Fig. 4a). According to the immunofluorescence staining of tumor cells, CRT was recruited from cytoplasm and aggregated on the plasma membrane; meanwhile, HMGB1 was translocated from the nucleus to the cytoplasm (Figs. 4b & S17). Thus, with Zn^2+^ as an artificial proinflammatory cytokine, BaSO_4_@ZIF-8/TRF NMΦs are able to trigger the immunogenic cell death of tumors to expose tumor antigens, which is similar to natural macrophages.

### Antigen Capture of BaSO_4_@ZIF-8/TRF NMΦs

In addition to cytokine secretion, antigen capture is another feature of macrophages [46]. After the decomposition of ZIF-8 shell in an acidic tumor condition, tremendous voids with average diameter of *c.a.* 20 nm were exposed on the BaSO_4_ nanoparticles (Figs. 4c & S18). Previous studies have confirmed that Ba possesses high affinity to cellular proteins, especially Ca-enriched membrane protein [47]. In many cases, Ba and Ca share the same binding sites of membrane proteins, thereby allowing Ba to substitute Ca for membrane protein binding [48]. Thus, BaSO_4_ nanoparticles are assumed to be capable of binding membrane proteins for capturing tumor associated antigens. The selective affinity of BaSO_4_@ZIF-8/TRF NMΦs to tumor cells was investigated by SEM. Due to the binding ability of Ba to membrane proteins, bare BaSO_4_ nanoparticles were recruited to the surface of 4T1 tumor cell or L02 normal cell, which is lack of selectivity (Fig. 4d). For BaSO_4_@ZIF-8 or BaSO_4_@ZIF-8/TRF NMΦs, the ZIF-8 shell blocked the direct interaction of Ba to membrane proteins, thus only a little of them was found on the surface of L02 cells. However, the ZIF-8 shell was selectively decomposed under tumor acidic microenvironment to recover the interaction of BaSO_4_ with membrane proteins. As a result, BaSO_4_@ZIF-8 or BaSO_4_@ZIF-8/TRF NMΦs are specifically bound to the surface of 4T1 tumor cells. Because of the specific affinity of TRF to tumor cells, the accumulation of BaSO_4_@ZIF-8/TRF NMΦs on the 4T1 tumor cell membrane was over twofold higher than that of BaSO_4_@ZIF-8 nanoparticles. To further assess the capturing selectivity, the binding capacity of BaSO_4_ nanoparticles to membrane proteins was quantified in the presence of serum proteins (*e.g.* bovine serum albumin, BSA). When exposed to physiological environments (pH 7.4), the binding ability of BaSO_4_@ZIF-8/TRF NMΦs to TRF is 63.8 mg g^−1^, but its binding ability to cell protein or serum protein is less than 12.0 mg g^−1^ (Fig. 4e). By contrast, under tumor acidic conditions, nearly 80% of TRFs were desorbed from BaSO_4_@ZIF-8/TRF NMΦs. Meanwhile, the binding capacity of BaSO_4_@ZIF-8/TRF NMΦs towards membrane proteins was increased to 44.9 mg g^−1^, much higher than that of serum proteins, suggesting that BaSO_4_@ZIF-8/TRF NMΦs exhibit selective enrichment of tumor cell proteins, which is similar to the selective capture of tumor antigens by natural macrophages. The selective binding of BaSO_4_ to membrane proteins (*e.g.* CRT and E-cadherin) was further investigated through molecular dynamics (MD) simulation. Either membrane proteins or serum proteins can interact with BaSO_4_ by van der Waals interactions and Coulomb force. However, the binding energy of BaSO_4_ with CRT and E-cadherin was about 1.6- and 2.4-fold lower than that of BaSO_4_ with serum protein, respectively (Figs. 4f & S19). Thus, BaSO_4_ nanoparticles possess specific binding ability to membrane proteins for capturing tumor antigens.

In the process of anoikis, the detached cancer cells are likely to migrate and recolonized, resulting in anoikis resistance and tumor metastasis [49]. To prevent cancer cell recolonization, various cell adhesion inhibitors have been developed, such as oligopeptides or small molecular complexes [50]. In this work, BaSO_4_ nanoparticles are demonstrated to be with high affinity to E-cadherin (Fig. 4g). Therefore, BaSO_4_ nanoparticles can act as a cell adhesion inhibitor to prevent cancer cell recolonization after ZIF-8/TRF-mediated anoikis. Our experimental results confirm that BaSO_4_ nanoparticles enhance tumor anoikis mediated by ZIF-8/TRF, although its cytotoxicity is weak (Fig. S10).

### Immune Activating Ability of BaSO_4_@ZIF-8/TRF NMΦ

**Fig. 5:**
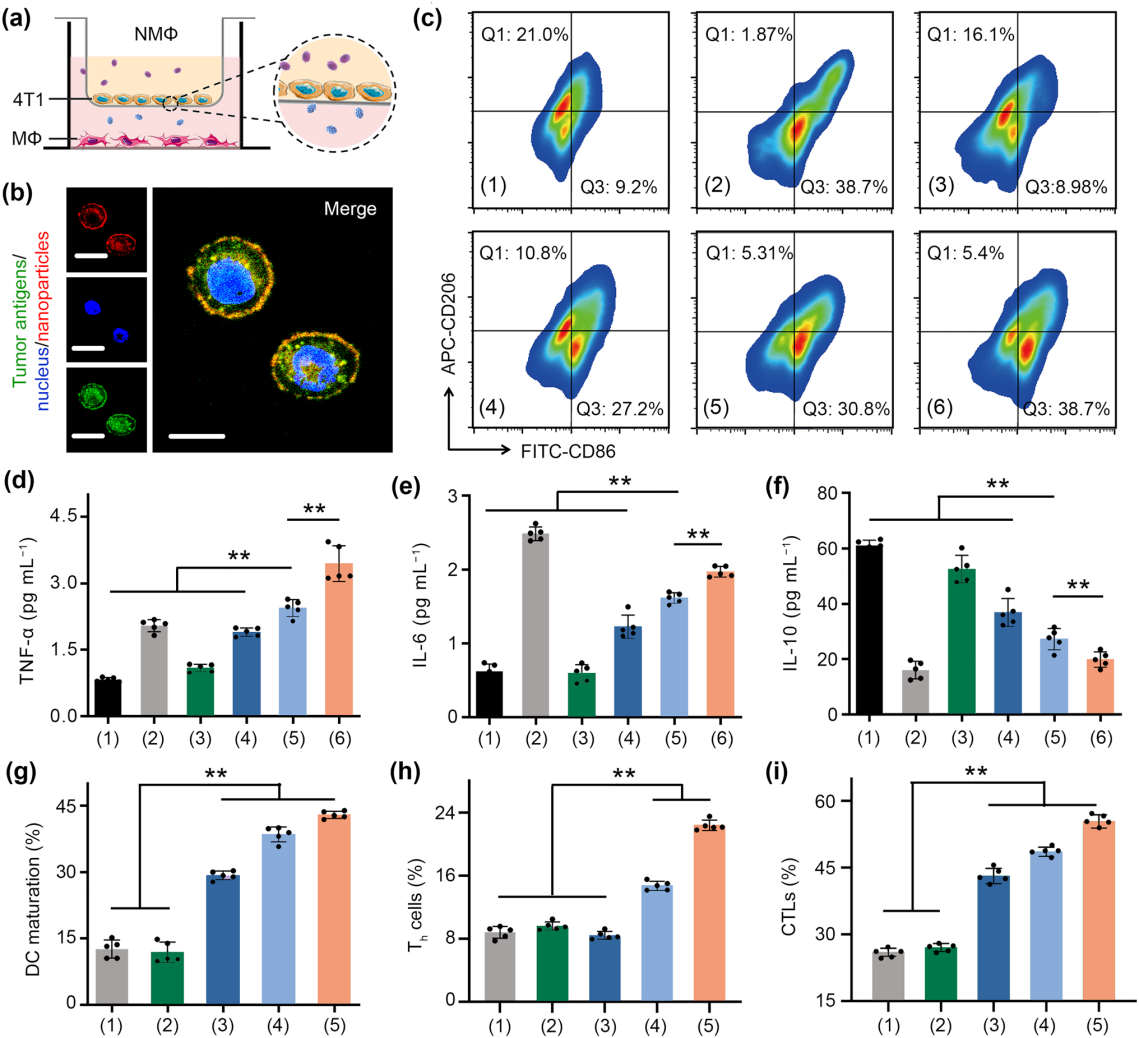
Antitumor immunity in vivo and in vitro. **a** Illustration of the transwell model to study the in vitro antigen capture of BaSO_4_@ZIF-8/TRF NMΦs. 4T1 tumor cells and J774A.1 macrophages (MΦ) were seeded in the upper and lower chambers of the transwells, respectively. **b** Confocal fluorescence images showing the locations of tumor antigens and nanoparticles in macrophages (scale bars = 15 μm). **c** Populations of M1 and M2 macrophages were analyzed by flow cytometry after different treatments: (1) control, (2) LPS, (3) BaSO_4_, (4) ZIF-8/TRF, (5) BaSO_4_@ZIF-8, (6) BaSO_4_@ZIF-8/TRF. The secretions of **d** TNF-α, **e** IL-6, and **f** IL-10 from J774.1A after different treatments. **g** The percentages of DC maturation, **h** populations of T helper cells (CD3^+^/CD4^+^, T_h_ cells), and **i** cytotoxic T cells (CD3^+^/CD8.^+^, CTLs) in vivo after different treatments: (1) control, (2) BaSO_4_, (3) ZIF-8/TRF, (4) BaSO_4_@ZIF-8, (5) BaSO_4_@ZIF-8/TRF. **p < 0.01 (n = 5)

As an artificial macrophage, the immune activating ability of BaSO_4_@ZIF-8/TRF NMΦs in vitro was examined via transwell invasion assay (Fig. 5a). For the treatment of ZIF-8/TRF without BaSO_4_ cores, less than 20% of macrophages phagocytized partial tumor antigens from the anoikis tumor cells (Fig. S20). Comparatively, after the treatment of BaSO_4_@ZIF-8/TRF NMΦs, tumor antigens were uptaken by more than 80% of macrophages (Fig. 5b). Moreover, with the addition of BaSO_4_@ZIF-8/TRF NMΦs, the population of M1 macrophage was elevated from 9.2% to 38.7%, significantly higher than that of ZIF-8/TRF (27.2%) and BaSO_4_@ZIF-8 (30.8%) (Figs. 5c & S21). Besides, the secretion of pro-inflammatory cytokines (*i.e.,* TNF-α and IL-6) approximately quadrupled in the presence of BaSO_4_@ZIF-8/TRF NMΦs; whereas the secretion of anti-inflammatory IL-10 was decreased by 67% (Fig. 5d–f). Therefore, BaSO_4_@ZIF-8/TRF NMΦ can mimic natural immunoactive macrophages to capture tumor antigens for activating immune response.

Next, we evaluated the ability of BaSO_4_@ZIF-8/TRF NMΦs to modulate immune cells in vivo. Since tumors are usually surrounded by tumor associated macrophages [51], BaSO_4_@ZIF-8/TRF NMΦs were intratumorally injected into mice to mimic the spacial distribution of natural macrophages. Owing to the specific affinity of TRF to tumor, the retention of BaSO_4_@ZIF-8/TRF NMΦs in tumor tissue was 25% higher than that in the BaSO_4_@ZIF-8 group (Fig. S22). The high affinity of TRF to tumors mimics the specific interactions of natural macrophages with tumors, endowing BaSO_4_@ZIF-8/TRF NMΦs with tumor resident ability [52]. The intratumoral Zn^2+^ level was examined by fluorescence staining of tumor slices with Zinquin as a Zn^2+^ indicator. Obviously, the Zn^2+^ level was remarkably increased about 4 times post injection of BaSO_4_@ZIF-8/TRF NMΦs (Fig. S23). BaSO_4_@ZIF-8/TRF NMΦs dramatically promoted the M1 macrophage rate from 4.65% to 28.3%, which was more efficient than ZIF-8/TRF nanoparticles (Fig. S24). Accompanying with M1 macrophage polarization, the serum levels of IL-6 and TNF-α were increased 11.5- and 2.2-fold by the NMΦs, respectively (Fig. S25); while the serum IL-10 level was decreased by 70%. Furthermore, the maturation rate of dendritic cells (CD11c^+^CD80^+^CD86^+^) was increased from 12.6% ± 2.1% to 42.6% ± 2.5% by the NMΦs (Figs. 5g & S26). For mice treated with BaSO_4_@ZIF-8/TRF, the population of CD8^+^ cytotoxic T lymphocytes and CD4^+^ T helper cells in spleens were individually increased by 30.2% ± 2.7% and 13.5% ± 2.1% (Figs. 5h–i & S27), much higher than that of other treatments. The recruitment of T cells within tumors was detected through immunohistochemistry staining. Because of the differentiation of T cells, the infiltration of CD4^+^ and CD8^+^ T cells in tumors was remarkably increased after the treatment of BaSO_4_@ZIF-8/TRF NMΦs (Fig. S28). IFN-γ as a prototypical antitumor cytokine was also overexpressed, indicating the immune activation by BaSO_4_@ZIF-8/TRF NMΦs (Fig. S29). FoxP3^+^ regulatory T cells (FoxP3^+^ Tregs) are a subset of T cells that can inhibit the immune system from attacking tumors to antagonize CD8^+^ cytotoxic T lymphocytes and CD4^+^ T helper cells. BaSO_4_@ZIF-8/TRF NMΦs decreased the population of FoxP3^+^ Tregs by nearly 80% (Fig. S30). Based on the above analysis, BaSO_4_@ZIF-8/TRF NMΦs successfully reversed tumor immunosuppression and activated systemic antitumor immunity.

### Reconstruction of Antitumor Immunity in vivo

**Fig. 6:**
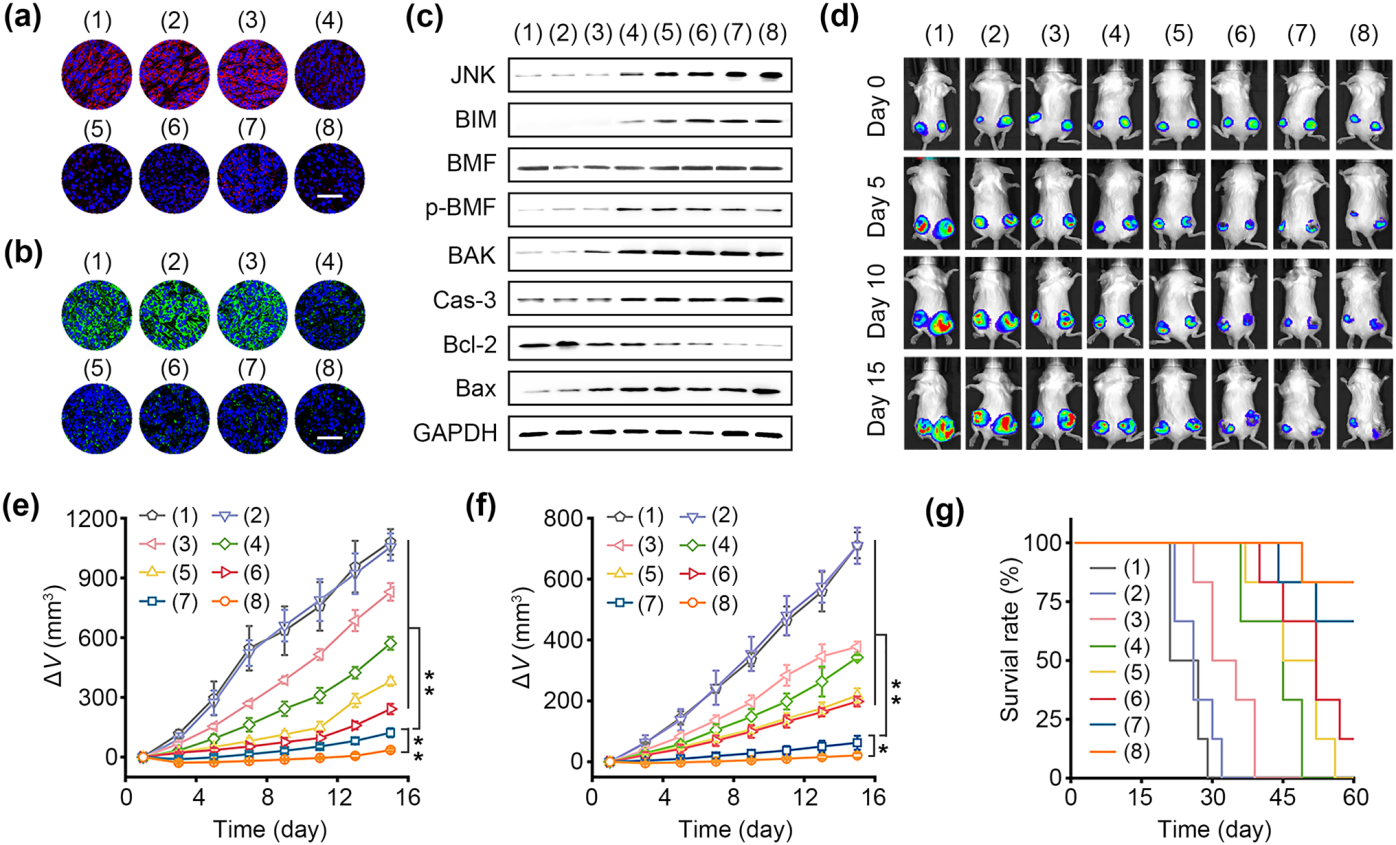
Antitumor therapeutic effect of NMΦ in vivo. **a** The immunofluorescence staining of E-cadherin (red fluorescence), and **b** integrin β1 (green fluorescence) in primary 4T1 tumor tissues after different treatments (scale bars: 100 μm). The nuclei were stained with blue fluorescence. **c** The expression of anoikis-related proteins in 4T1 tumors was analyzed through Western blotting after different treatments. **d** Representative bioluminescence images of 4T1-Luc tumor-bearing mice in different groups. **e** Growth curves of primary and **f** distant tumors in mice of different groups. **g** Survival curves of mice of different groups. Groups: (1) control, (2) BaSO_4_, (3) αPD-1, (4) ZIF-8/TRF, (5) BaSO_4_@ZIF-8, (6) BaSO_4_@ZIF-8/TRF, (7) ZIF-8/TRF + αPD-1, (8) BaSO_4_@ZIF-8/TRF + αPD-1. *p < 0.05, **p < 0.01 (n = 6)

The outstanding performance of BaSO_4_@ZIF-8/TRF NMΦs encourages us to evaluate their therapeutic efficiency in bilateral 4T1 tumor-bearing mice. To suppress immune tolerance of tumors, PD-1 antibody as a typical immune checkpoint blockade agent was administrated during the treatments. Upon injection of BaSO_4_@ZIF-8/TRF NMΦs, the expressions of adhesion-related proteins (*i.e.,* integrin β1 and E-cadherin) were sharply downregulated (Fig. 6a and b). Notably, the adhesion-related proteins were not significantly varied after treatment with αPD-1 or BaSO_4_ alone. To confirm tumor anoikis in vivo, the expressions of anoikis-related proteins were determined via Western blotting assay (Figs. 6c & S31). After the treatment with ZIF-8/TRF, BaSO_4_@ZIF-8 and BaSO_4_@ZIF-8/TRF, the JNK signaling pathway was activated to promote BMF phosphorylation and BIM expression. Subsequently, BAX/BAK pathway was involved to mediate the anoikis, accompanied with the downregulation of Bcl-2 and overexpression of caspase-3. Therefore, BaSO_4_@ZIF-8/TRF NMΦ can elicit tumor anoikis by virtue of Zn^2+^ release, which is similar to the cytotoxic process of macrophages via cytokine secretion.

Tumor growth was continuously monitored by bioluminescence imaging (Fig. 6d). Obviously, both the primary or distant tumors grew rapidly regardless of the treatment with anti-PD-1 alone or BaSO_4_ nanoparticles, indicating their faint antitumor activity (Fig. 6e and f). By contrast, for mice treated with BaSO_4_@ZIF-8/TRF NMΦs, the bioluminescence intensity and tumor volumes of primary tumors were remarkably reduced by 70% on day 15 (Fig. S32). The high antitumor efficacy of BaSO_4_@ZIF-8/TRF NMΦs could be ascribed to tumor anoikis. When anti-PD-1 was co-administrated with BaSO_4_@ZIF-8/TRF NMΦs, the tumor suppression rate further reached 95% due to the activation of antitumor immunity. Moreover, cytotoxic T cells and helper T cells were able to migrate into distant tumors for immunological attack, thereby inhibiting the growth of distant tumors by 90% (Fig. S33). After the treatment of BaSO_4_@ZIF-8/TRF NMΦs with anti-PD-1, obvious nucleus condensation and DNA fragmentations of dead cells were found from histological analysis of tumor slices (Figs. S34 & S35). Because of the immune modulating role of BaSO_4_@ZIF-8/TRF NMΦs, the mice survival rate was substantially increased to 83% within 60 days (Fig. 6g). During the treatments, all the mice behaved normally without significant loss of body weight (Fig. S36).

**Fig. 7:**
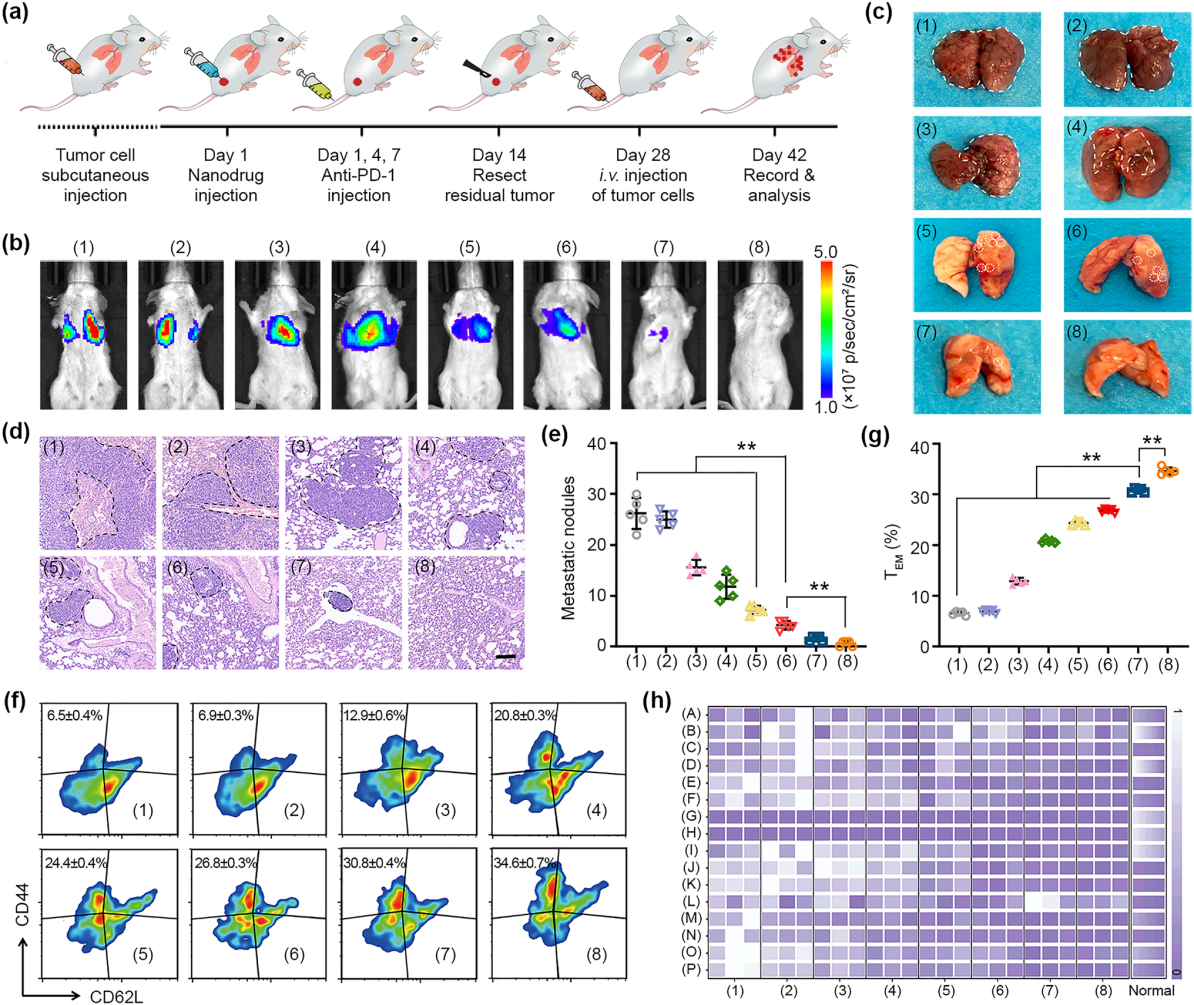
Anti-lung metastasis and immune memory effect of NMΦ in vivo. **a** Schedule for the in vivo studies of immune memory effect. **b** In vivo bioluminescence images of lung metastasis after different treatments. **c** Digital photos of metastatic nodes in the lungs with different treatments. **d** Lungs were examined through H&E staining on day 42 (scale bar = 100 μm). **e** Statistical results of metastatic nodules in the lungs. **f** Flow cytometry analysis of effector memory T cells (T_EM_) in splenic lymphocytes (gating on CD8.^+^), and **g** corresponding statistical results after different treatments. **h** Blood biochemistry analysis of mice in different groups: **A** white blood cell; **B** lymphocyte; **C** mononuclear cell; **D** neutrophile granulocyte; **E** hemoglobin; **F** red blood cell; **G** hematocrit; **H** mean corpuscular volume; **I** mean corpuscular hemoglobin; **J** mean corpuscular hemoglobin concentration; **K** red blood cell distribution width-standard deviation; **L** red blood cell volume distribution width the coefficient; **M** platelet count; **N** platelet crit; **O** mean platelet volume; **P** platelet distribution width. Groups: (1) control, (2) BaSO_4_, (3) αPD-1,(4) ZIF-8/TRF, (5) BaSO_4_@ZIF-8, (6) BaSO_4_@ZIF-8/TRF, (7) ZIF-8/TRF + αPD-1, (8) BaSO_4_@ZIF-8/TRF + αPD-1. **p < 0.01 (n = 5)

To evaluate the immune memory effect, tumor-bearing mice were rechallenged with intravenous injection of 4T1-Luc tumor cells after various treatments (Fig. 7a). For blank and BaSO_4_ groups, bright bioluminescence was detected in the chest area on day 42, and tremendous metastatic nodules were distributed over 75% of the lung surface. In mice treated with BaSO_4_@ZIF-8/TRF and anti-PD-1, the bioluminescence signal of tumor metastasis was almost undetectable (Fig. 7b), and lung metastasis was completely suppressed (Fig. 7c–e). Flow cytometry analysis reveals fivefold increase of the population of effector memory T cells (T_EM_) in the group of BaSO_4_@ZIF-8/TRF + anti-PD-1. Therefore, BaSO_4_@ZIF-8/TRF NMΦs can successfully elicit the long-term immune memory effect to defend against tumor recurrence (Fig. 7f and g). According to the H&E staining results, the main organs kept normal histological structures (Fig. S37). Moreover, the blood biochemistry indexes were within the normal ranges (Fig. 7h). BaSO_4_@ZIF-8/TRF NMΦs can be metabolized by the body within 72 h even after intravenous injection into the mice (Fig. S38). All these aspects suggest the high biosafety of BaSO_4_@ZIF-8/TRF NMΦs for practical applications.

## Conclusions

In summary, with BaSO_4_@ZIF-8/TRF as a paradigm, we present an artificial macrophage to mimic macrophage-tumor interactive pattern for antitumor immune activation. BaSO_4_@ZIF-8/TRF NMΦs are able to selectively accumulate surrounding tumor cells and gradually release Zn^2+^ into tumor microenvironment. Zn^2+^ as a chemical messenger depressed the expression of adhesion proteins on cell membranes to induce tumor immunogenic death via anoikis. As the disintegration of ZIF-8 shell, a large number of mesopores were left on the BaSO_4_ nanoparticles, providing cavities for capturing tumor antigens. As a result, BaSO_4_ nanoparticles bearing tumor antigens promote the macrophage polarization and subsequent T cell recruitment for immune activation. Together with immune checkpoint inhibitors, BaSO_4_@ZIF-8/TRF NMΦ can elicit systemic antitumor immunity against distant metastasis. Moreover, the immune memory effect is successfully achieved to prevent tumor relapse. Overall, the BaSO_4_@ZIF-8/TRF NMΦ successfully simulates the basic biological functions of natural macrophages, including tumor retention, cytokine release, antigen capture and immune activation. The artificial macrophages with hierarchical nanostructure provide a promising strategy to overcome tumor immunosuppression, and raise the prospect of artificial cells in the biomedical field.

### Supplementary Information

Below is the link to the electronic supplementary material.

Supplementary file1 (PDF 3019 KB)
