# Supplementary material for: Artificial Macrophage with Hierarchical Nanostructure for Biomimetic Reconstruction of Antitumor Immunity
Source: Nanomicro Lett. 2023 Sep 22;15:216. doi: 10.1007/s40820-023-01193-4 (PMC10516848; doi:10.1007/s40820-023-01193-4)
Supplement: Supplementary file 1 — Supplementary file1 (PDF 3019 KB) [file 40820_2023_1193_MOESM1_ESM.pdf]

Supporting Information for

Artificial Macrophage with Hierarchical Nanostructure for Biomimetic Reconstruction of Antitumor Immunity

Henan Zhao[1], Renyu Liu[2], Liqiang Wang[3], Feiying Tang[4], Wansong Chen[1,*] and You-Nian Liu[1,*]

[1] Hunan Provincial Key Laboratory of Micro & Nano Materials Interface Science, College of Chemistry and Chemical Engineering, Central South University, Changsha, Hunan 410083, P. R. China

[2] Xiangya Hospital, Central South University, Changsha, Hunan 410008, P. R. China

[3] Henan Province Industrial Technology Research Institute of Resources and Materials, School of Material Science and Engineering, Zhengzhou University, Zhengzhou, Henan 450001, P. R. China

[4] College of Chemical Engineering, Xiangtan University, Xiangtan 411105, Hunan, P. R. China

*Corresponding authors. E-mail: chenws@csu.edu.cn (Wansong Chen); liuyounian@csu.edu.cn (You-Nian Liu)

Supplementary Figures and Tables

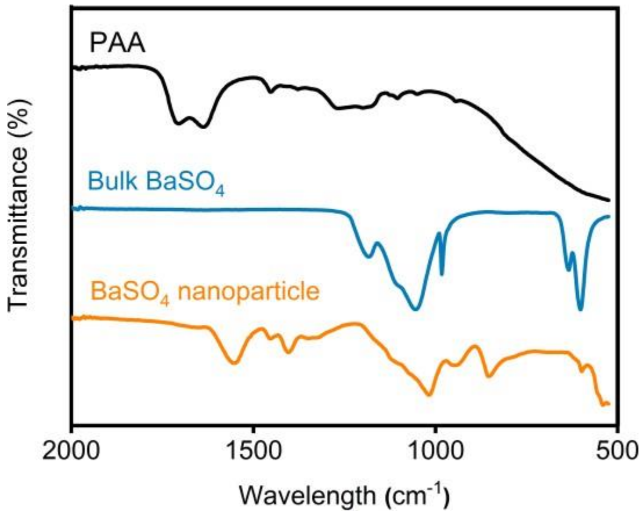

Fig. S1 FT-IR spectra of PAA, standard $BaSO_4$ and PAA-modified $BaSO_4$ nanoparticles

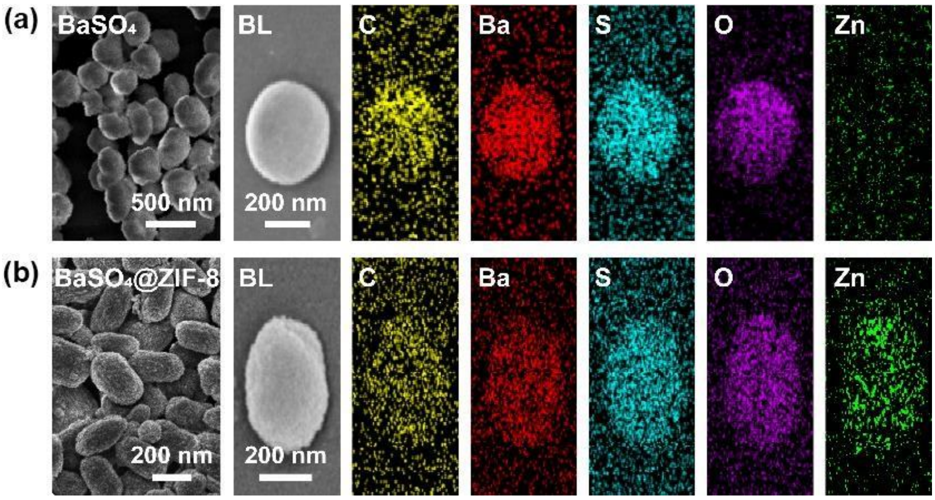

Fig. S2 SEM images and corresponding element mapping of **(a)** $BaSO_4$, **(b)** $BaSO_4$@ZIF-8

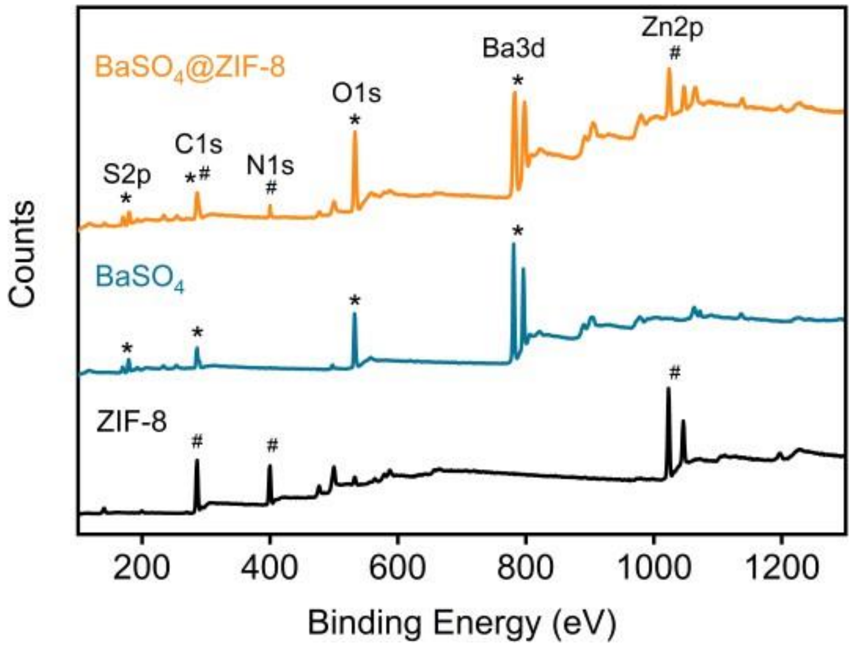

Fig. S3 XPS survey spectra of ZIF-8, $BaSO_4$ and $BaSO_4$@ZIF-8 nanoparticles

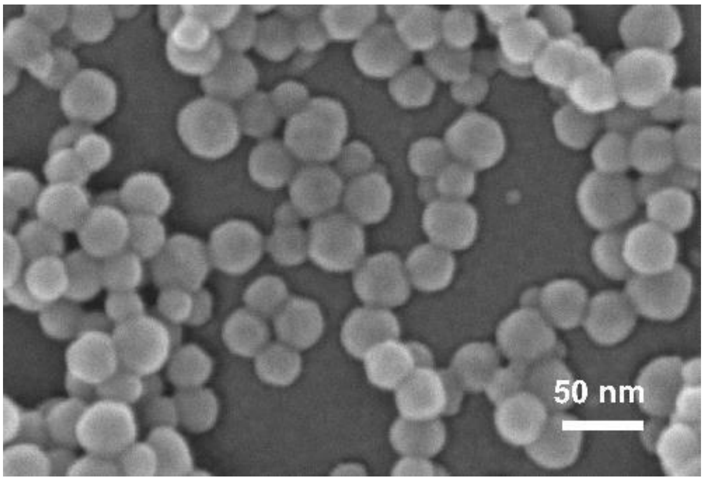

Fig. S4 SEM image of ZIF-8 nanoparticles

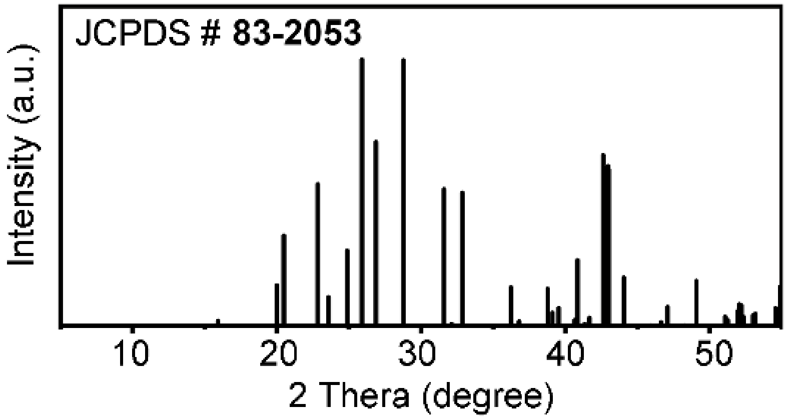

Fig. S5 Standard XRD pattern of $BaSO_4$

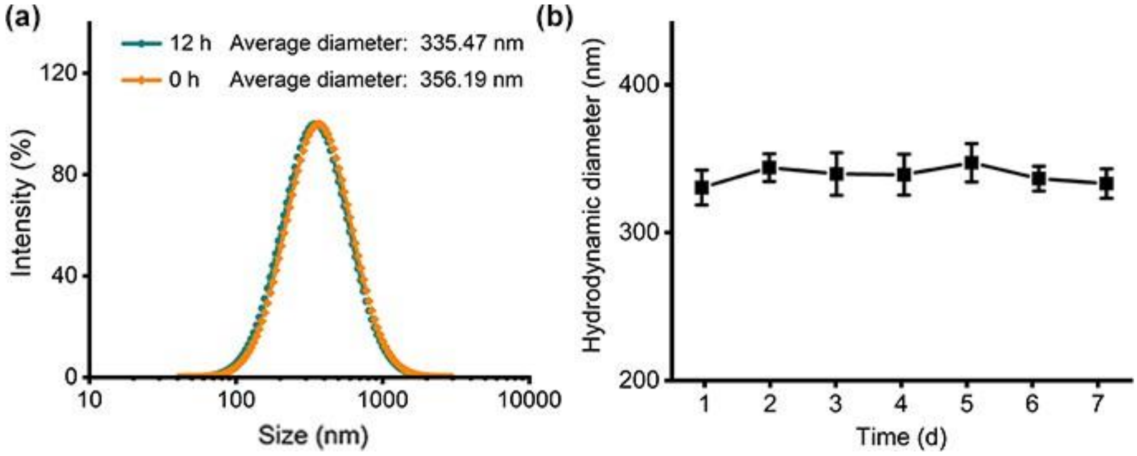

Fig. S6 (a) Hydrodynamic size distribution of $BaSO_4$@ZIF-8 nanoparticles at 0 h and 12 h in physiological saline. **(b)** The dispersion stability of $BaSO_4$@ZIF-8 nanoparticles in physiological saline within 7 days

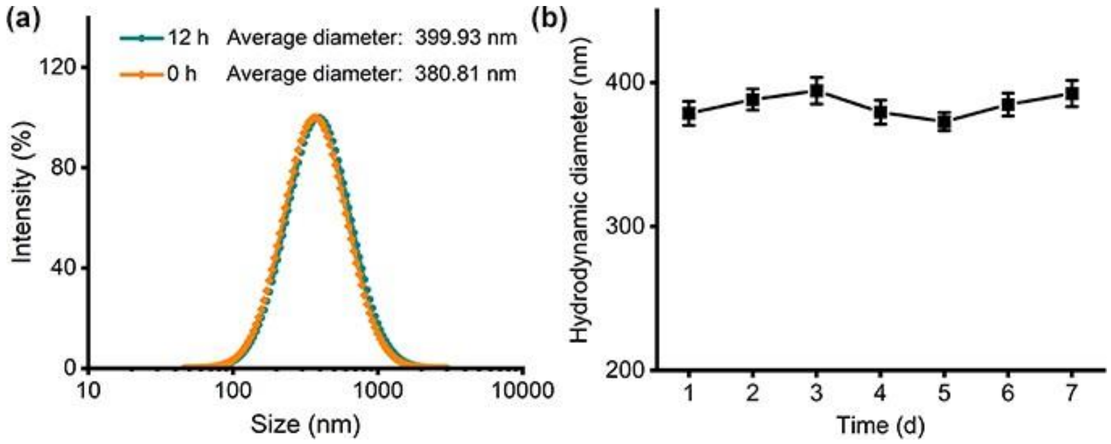

Fig. S7 (a) Average diameters of $BaSO_4$@ZIF-8/TRF NMΦs at 0 h, 12 h in physiological saline and **(b)** its dispersion stability within 7 days

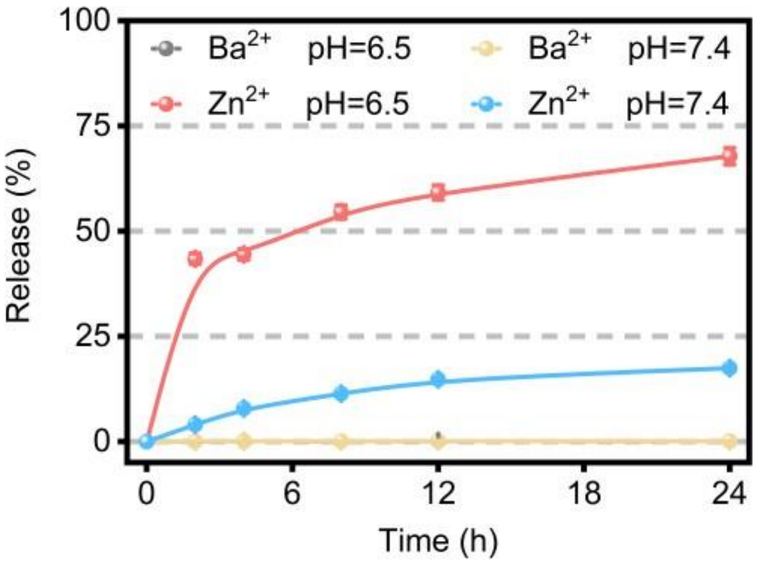

Fig. S8 Release of Ba^{2+} and Zn^{2+} from $BaSO_4$@ZIF-8/TRF NMΦs in the culture media of normal cells (pH 7.4) and tumor cells (pH 6.5)

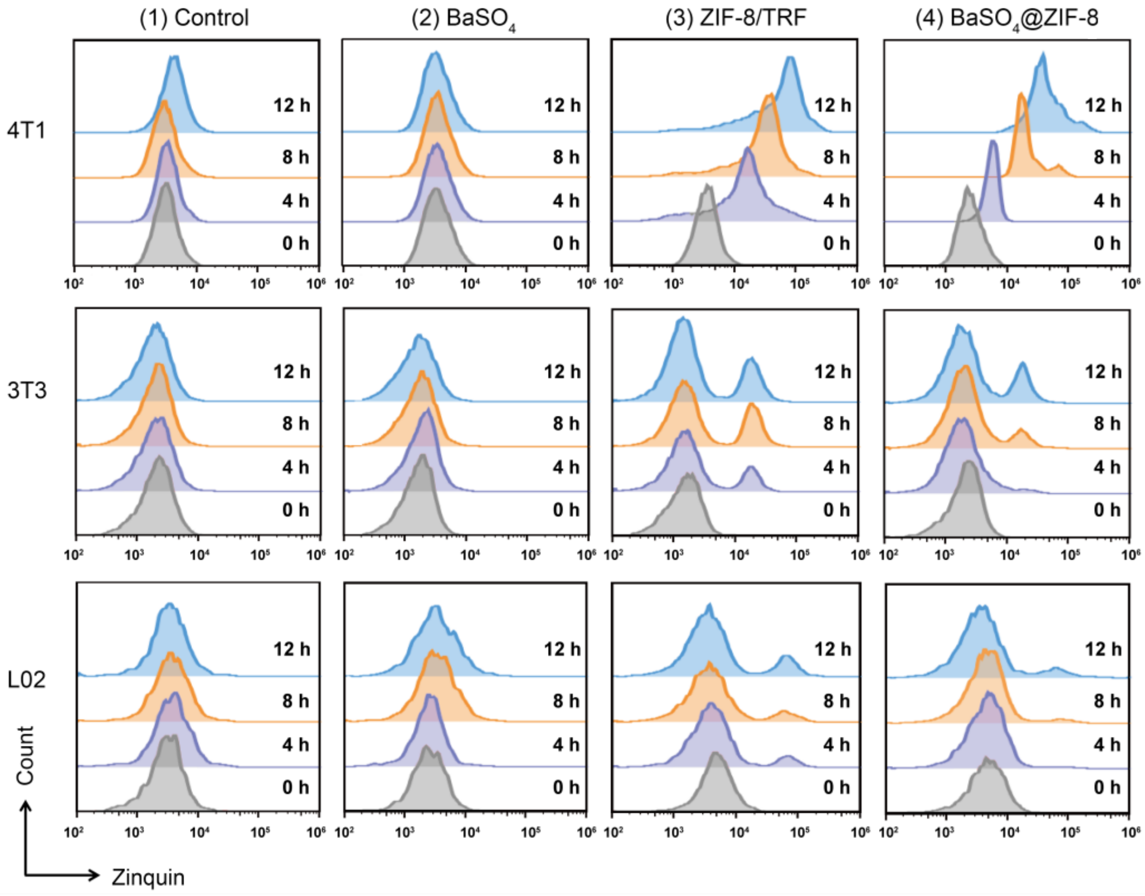

Fig. S9 Intracellular Zn^{2+} content of 4T1, 3T3 and L02 cells after incubation with $BaSO_4$, ZIF- 8/TRF, and $BaSO_4$@ZIF-8 for different time periods

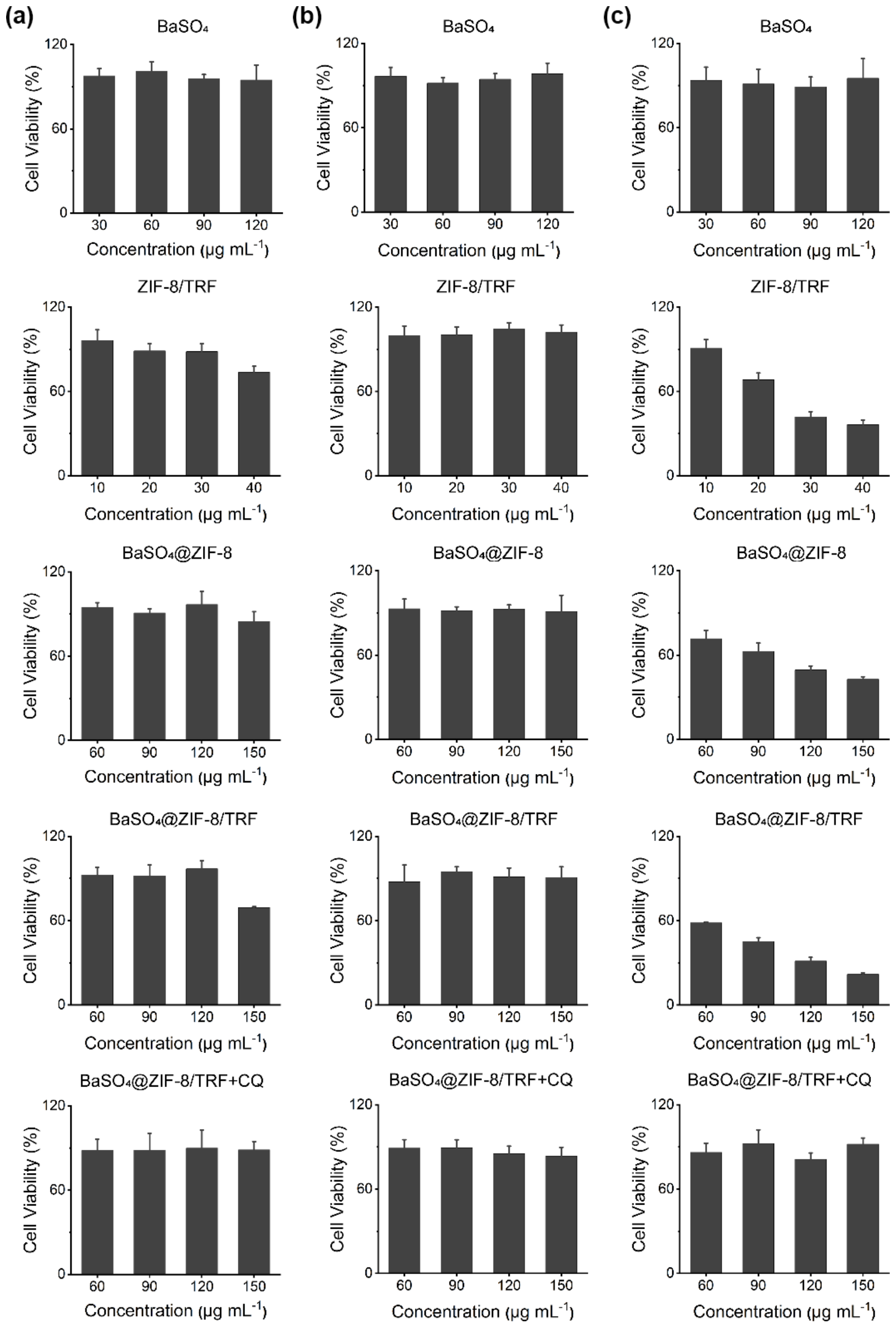

Fig. S10 Cell viability of **(a)** 3T3, **(b)** L02 and **(c)** 4T1 cells incubated with $BaSO_4$, ZIF-8/TRF, $BaSO_4$@ZIF-8, $BaSO_4$@ZIF-8/TRF and $BaSO_4$@ZIF-8/TRF + CQ

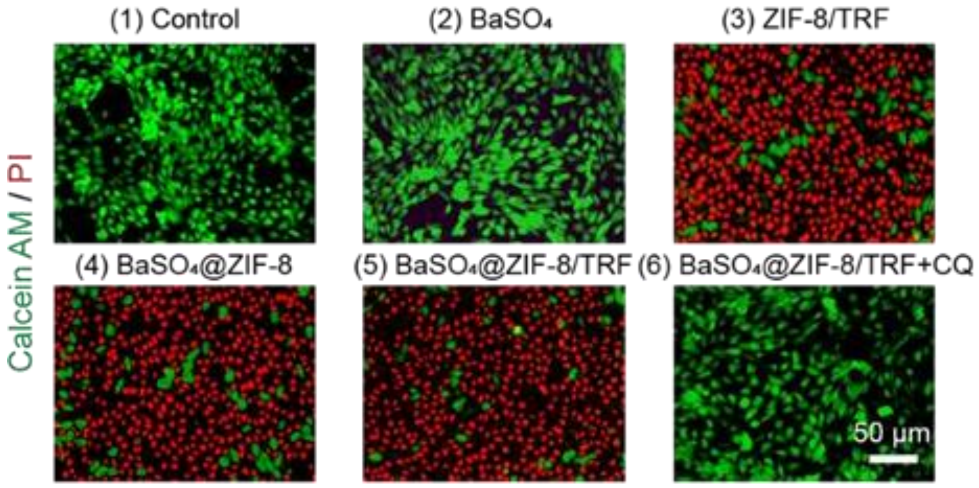

Fig. S11 Live-dead staining images of tumor cells after incubation with $BaSO_4$ (70 μg mL^{-1}), ZIF-8/TRF (30 μg mL^{-1}), $BaSO_4$@ZIF-8 (150 μg mL^{-1}), $BaSO_4$@ZIF-8/TRF (150 μg mL^{-1}), $BaSO_4$@ZIF-8/TRF (150 μg mL^{-1}) + CQ (3 μg mL^{-1}) for 24 h

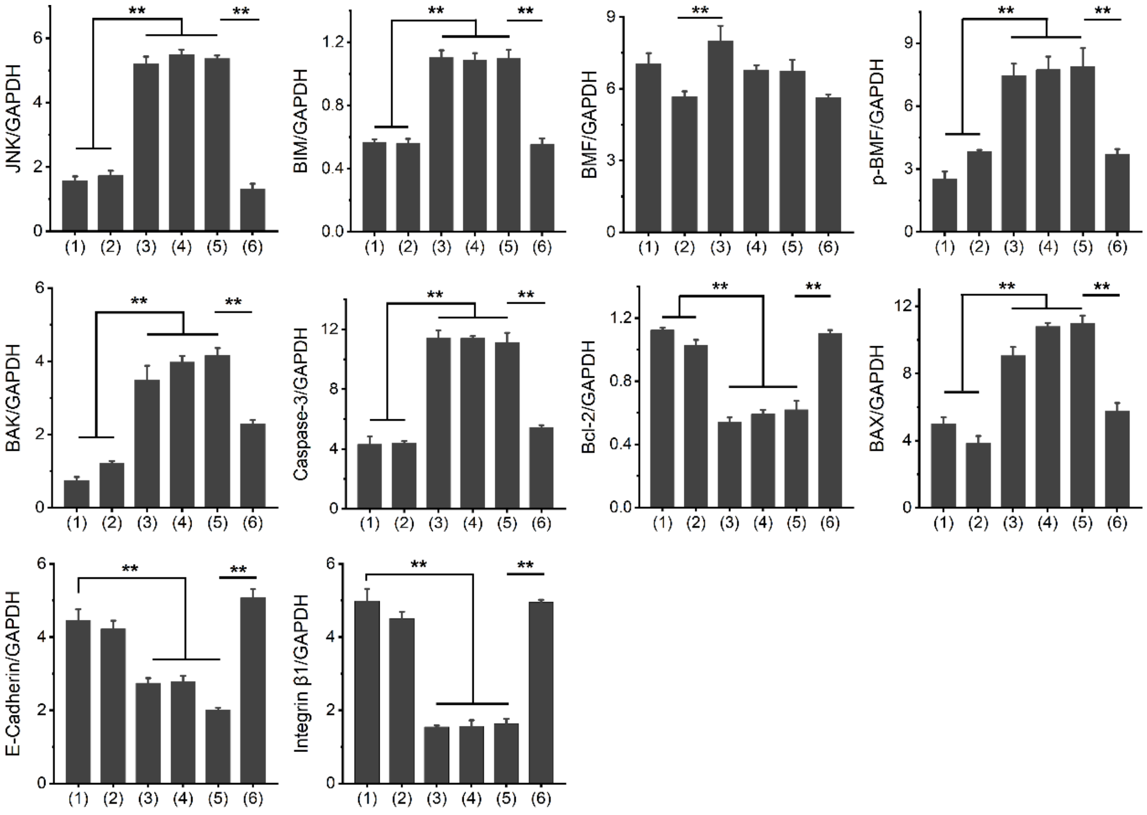

Fig. S12 The expression of anoikis-related proteins in 4T1 tumor cells after different treatments. Groups: (1) control; (2) $BaSO_4$; (3) ZIF-8/TRF; (4) $BaSO_4$@ZIF-8; (5) $BaSO_4$@ZIF-8/TRF; (6) $BaSO_4$@ZIF-8/TRF + CQ. $^{**}p < 0.01$ (n = 3)

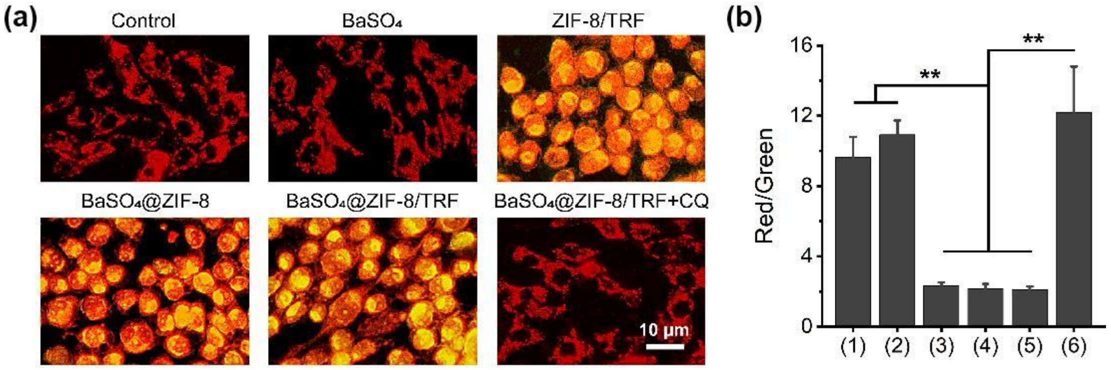

Fig. S13 (a) JC-1 staining of 4T1 tumor cells after different treatments. **(b)** Red-to-green fluorescence ratio of tumor cells in different groups. Groups: (1) control; (2) $BaSO_4$; (3) ZIF- 8/TRF; (4) $BaSO_4$@ZIF-8; (5) $BaSO_4$@ZIF-8/TRF; (6) $BaSO_4$@ZIF-8/TRF + CQ. **$p < 0.01$

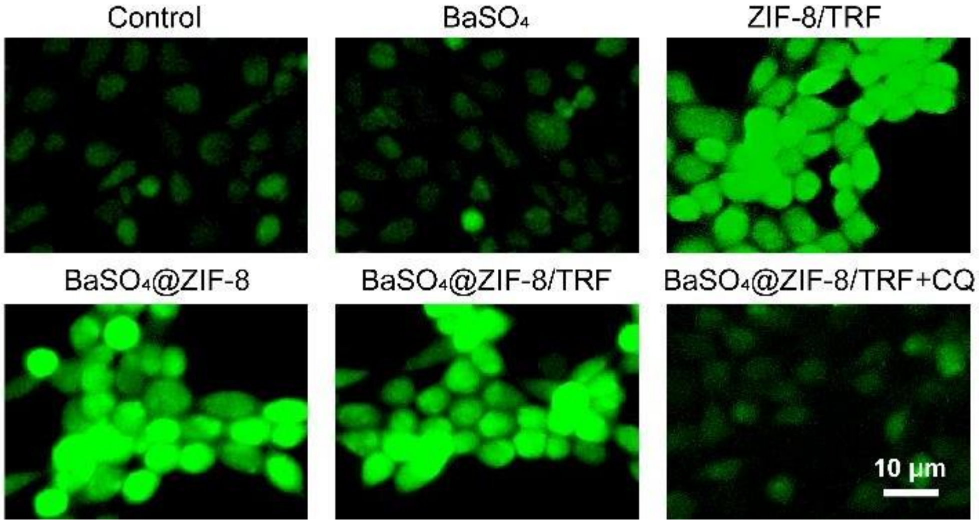

Fig. S14 Intracellular oxidative stress of 4T1 tumor cells after different treatments was examined through DCFH-DA staining

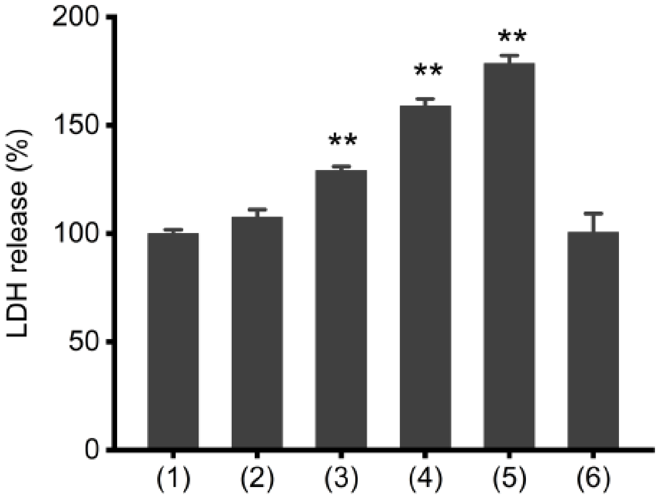

Fig. S15 The LDH release of tumor cells after different treatments for 24 h. Groups: (1) control; (2) $BaSO_4$; (3) ZIF-8/TRF; (4) $BaSO_4$@ZIF-8; (5) $BaSO_4$@ZIF-8/TRF; (6) $BaSO_4$@ZIF-8/TRF + CQ. **$p < 0.01$ (compared to the control group; n = 3)

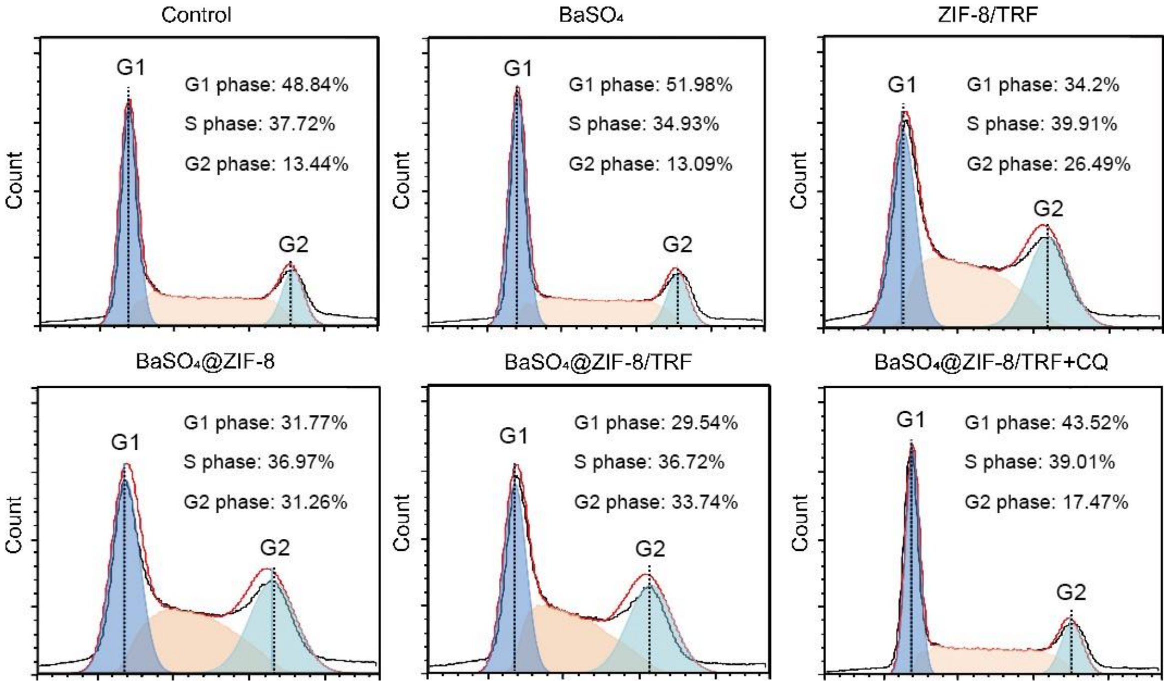

Fig. S16 Cell cycle distributions of tumor cells after different treatments

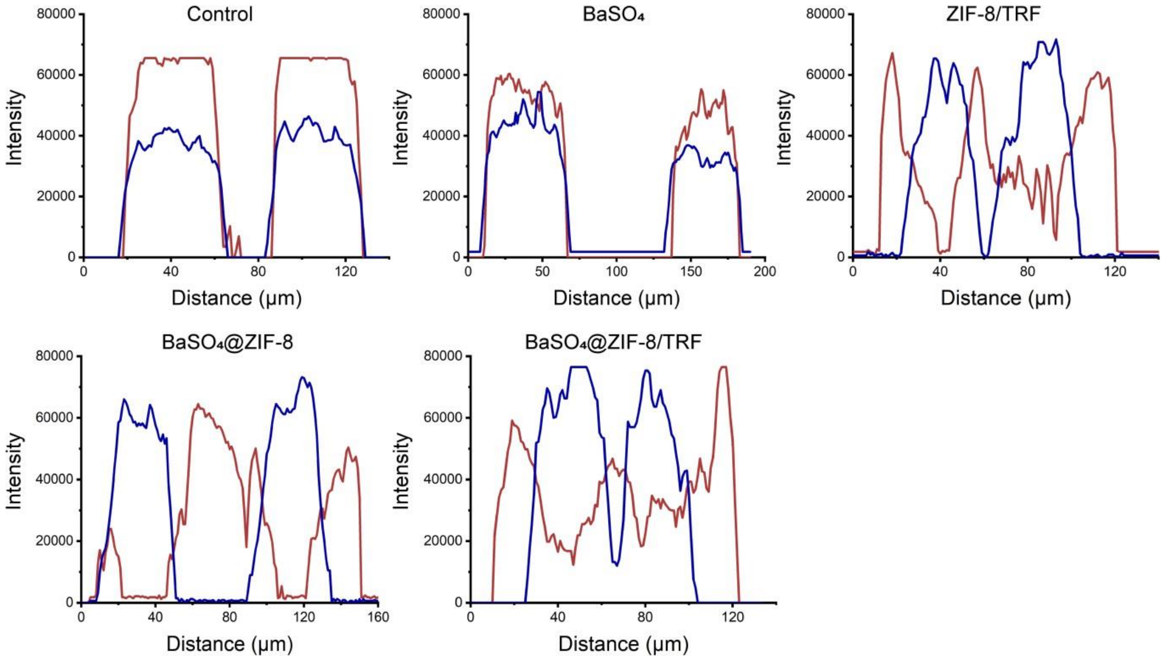

Fig. S17 Fluorescence distributions of HMGB1 (red line) and DAPI (blue line) within 4T1 tumor cells along the dash lines in Fig. 4B

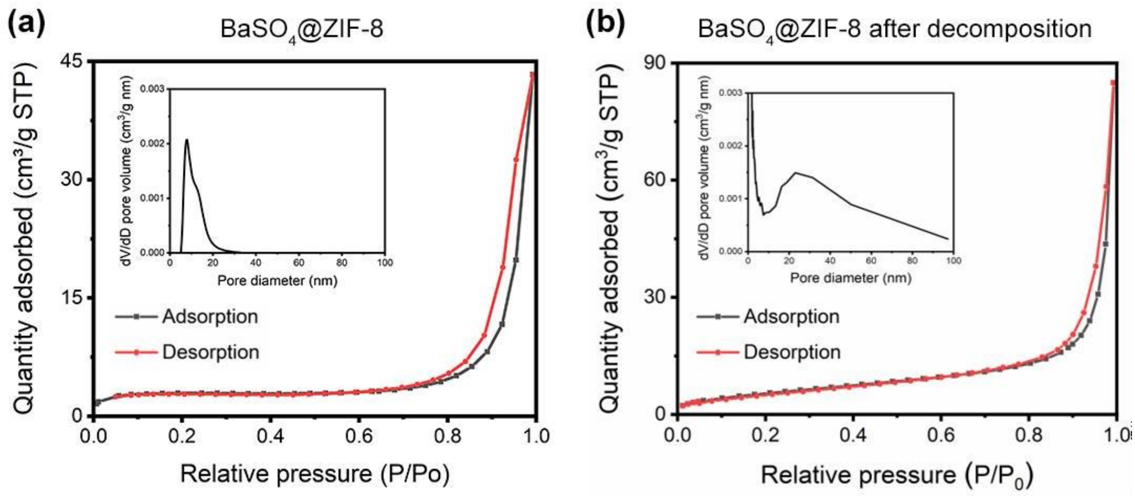

Fig. S18 Nitrogen adsorption/desorption isotherms and pore size distribution of **(a)** $BaSO_4$@ZIF-8 nanoparticles and **(b)** decomposed $BaSO_4$@ZIF-8 nanoparticles

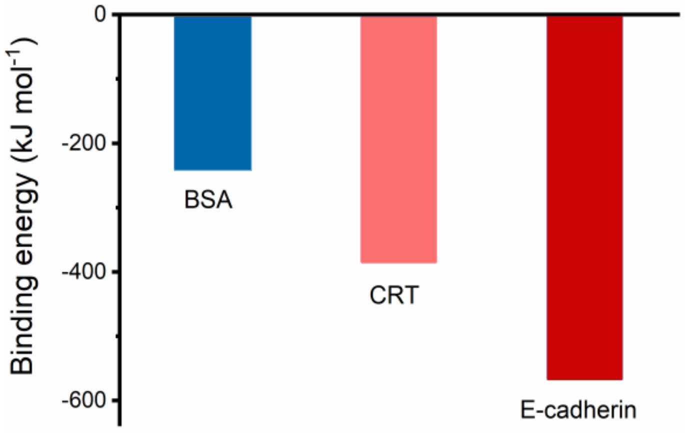

Fig. S19 Binding energy of different proteins on $BaSO_4$ nanoparticles

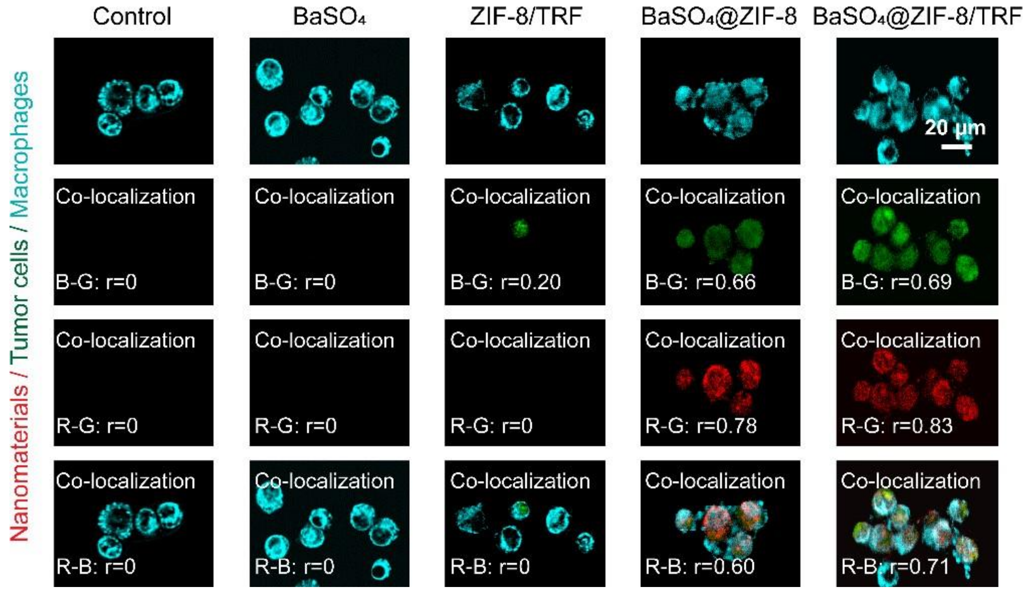

Fig. S20 Confocal fluorescence images showing the locations of tumor antigens and nanoparticles in macrophages

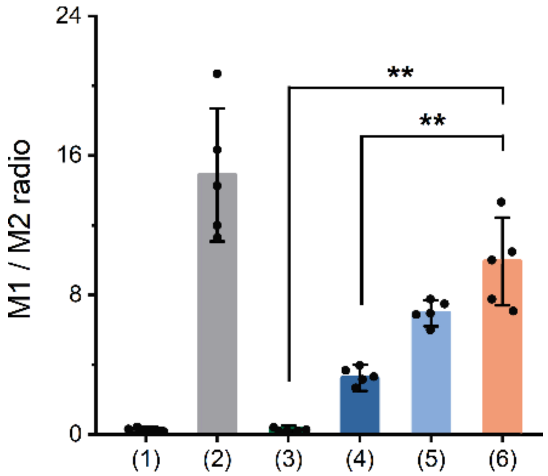

Fig. S21 Statistical results of macrophage polarization *in vitro* under different treatments: (1) control; (2) LPS; (3) $BaSO_4$; (4) ZIF-8/TRF; (5) $BaSO_4$@ZIF-8; (6) $BaSO_4$@ZIF-8/TRF (**$p < 0.01$, n = 5)

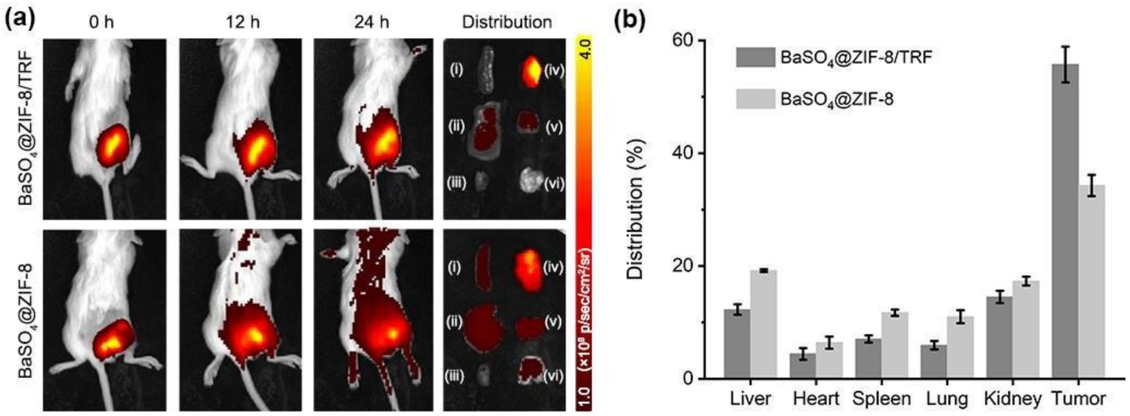

Fig. S22 (a) Near-infrared fluorescence images and **(b)** biodistribution results of $BaSO_4$@ZIF- 8 and $BaSO_4$@ZIF-8/TRF in 4T1 tumor-bearing post intratumoral injection (i: spleen; ii: liver; iii: heart; iv: tumor; v: kidney; vi: lung)

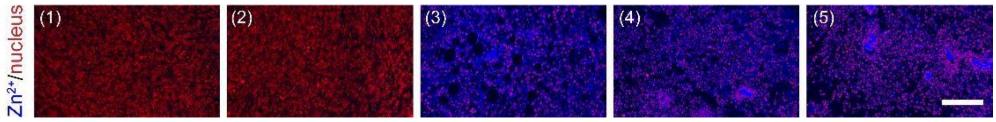

Fig. S23 Intratumoral Zn^{2+} was detected with Zinquin as a probe (blue fluorescence) after different treatments (scale bar = 100 μm). Groups: (1) PBS; (2) $BaSO_4$; (3) ZIF-8/TRF; (4) $BaSO_4$@ZIF-8; (5) $BaSO_4$@ZIF-8/TRF

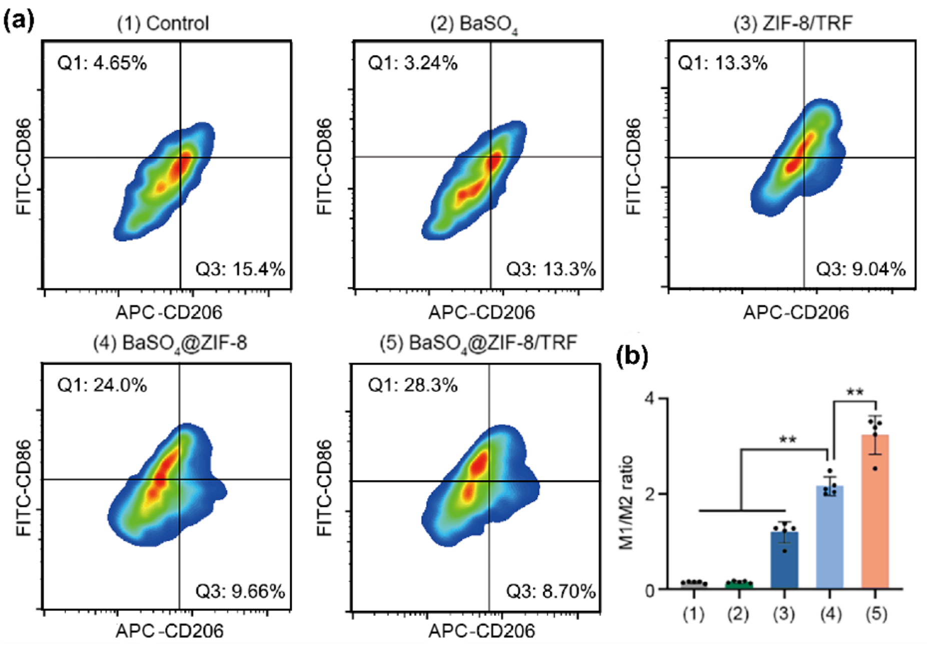

Fig. S24 (a) Flow cytometry data and **(b)** statistical results of M1 and M2 polarization *in vivo* after different treatments (**$p < 0.01$, n = 5)

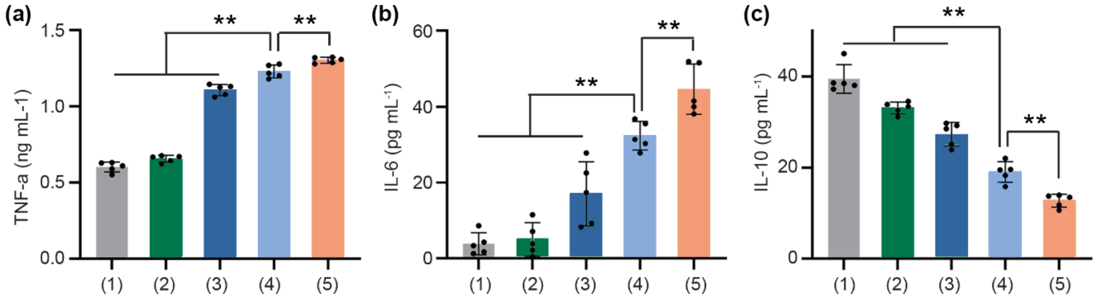

Fig. S25 (a) Serum TNF-α, **(b)** IL-6, and **(c)** IL-10 levels in tumor-bearing mice after different treatments. Groups: (1) control, (2) $BaSO_4$, (3) ZIF-8/TRF, (4) $BaSO_4$@ZIF-8, (5) $BaSO_4$@ZIF-8/TRF (**$p < 0.01$, n = 5)

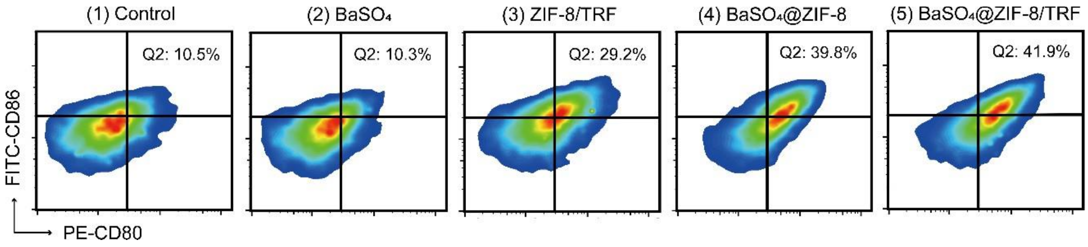

Fig. S26 DC maturation was analyzed by flow cytometry after different treatments (gating on $CD11c^+$)

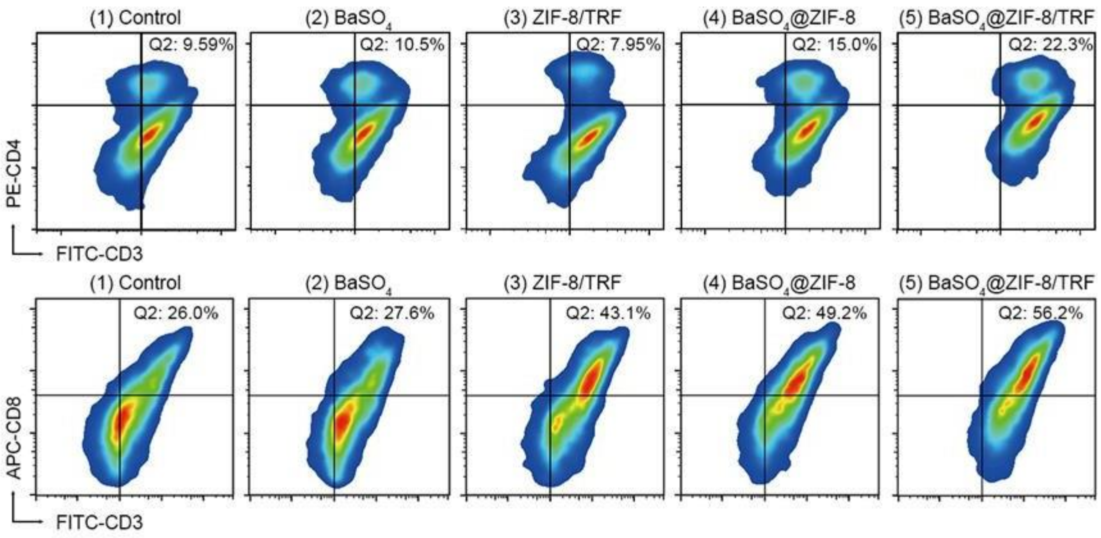

Fig. S27 Flow cytometry data of T helper cells ($CD3^+/CD4^+$, T_h cells) and cytotoxic T cells ($CD3^+/CD8^+$, CTLs) *in vivo* after different treatments

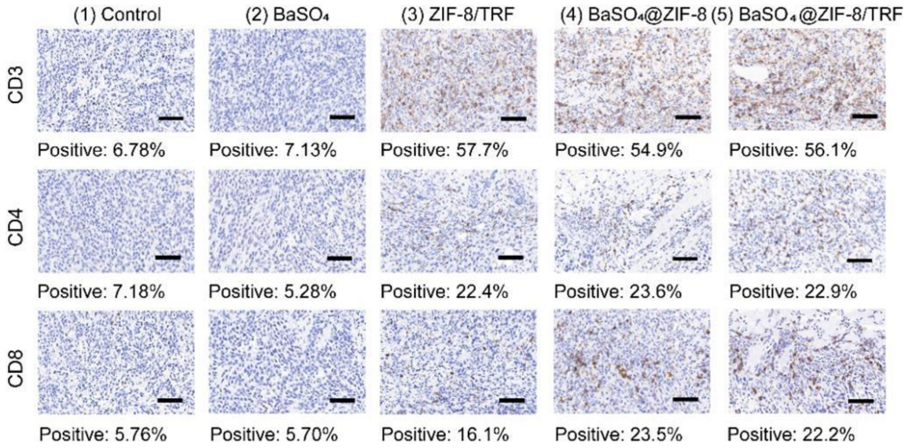

Fig. S28 The immunohistochemistry staining images of distant 4T1 tumor tissues after different treatments (scale bars = 100 μm)

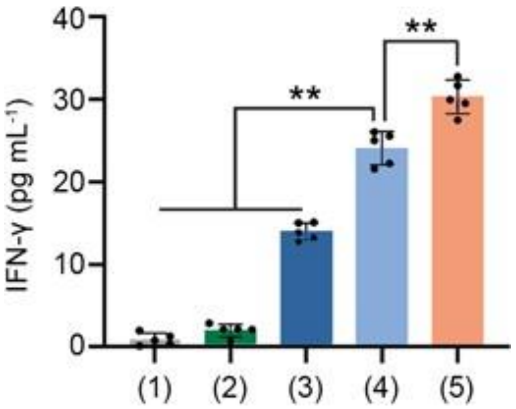

Fig. S29 Serum IFN-γ of mice after various treatments: (1) control, (2) $BaSO_4$, (3) ZIF-8/TRF, (4) $BaSO_4$@ZIF-8, (5) $BaSO_4$@ZIF-8/TRF (***p* < 0.01, n = 5)

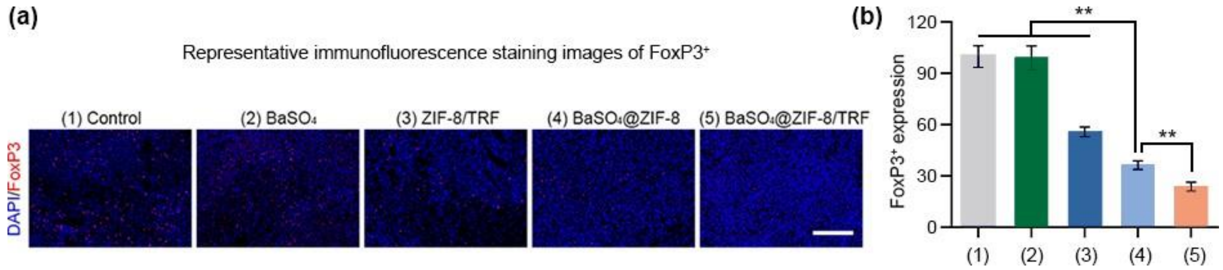

Fig. S30 (a) Representative immunofluorescence staining images of distant tumors and **(b)** quantitative results after different treatments. Scale bar = 100 μm. Groups: (1) control, (2) $BaSO_4$, (3) ZIF-8/TRF, (4) $BaSO_4$@ZIF-8, (5) $BaSO_4$@ZIF-8/TRF (**$p < 0.01$, n = 5)

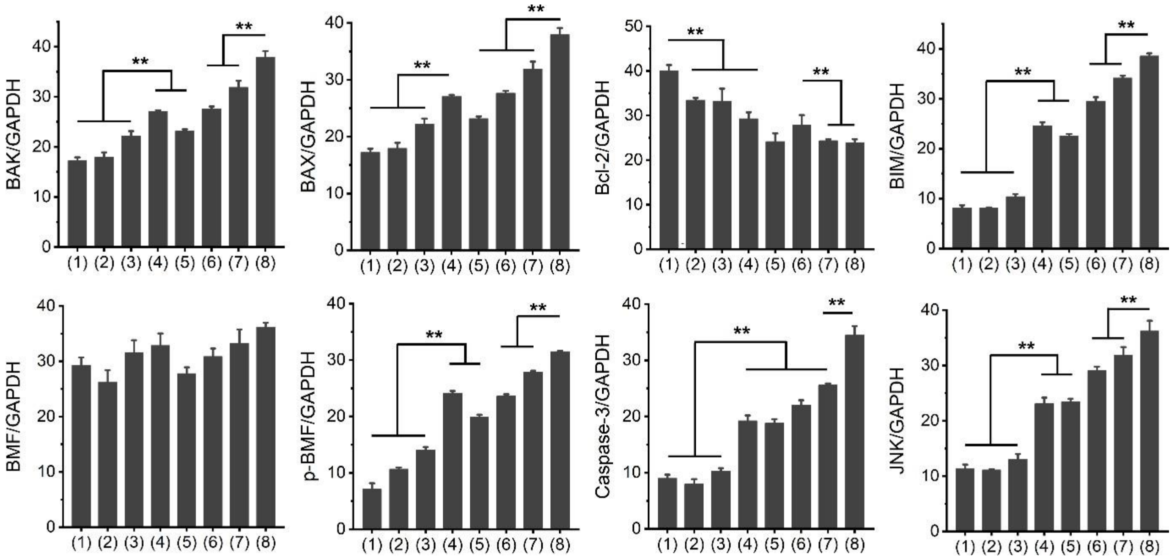

Fig. S31 The expression of anoikis-related proteins in tumors after different treatments (**$p < 0.01$). Group: (1) control, (2) $BaSO_4$, (3) αPD-1,(4) ZIF-8/TRF, (5) $BaSO_4$@ZIF-8, (6) $BaSO_4$@ZIF-8/TRF, (7) ZIF-8/TRF + αPD-1 and (8) $BaSO_4$@ZIF-8/TRF + αPD-1

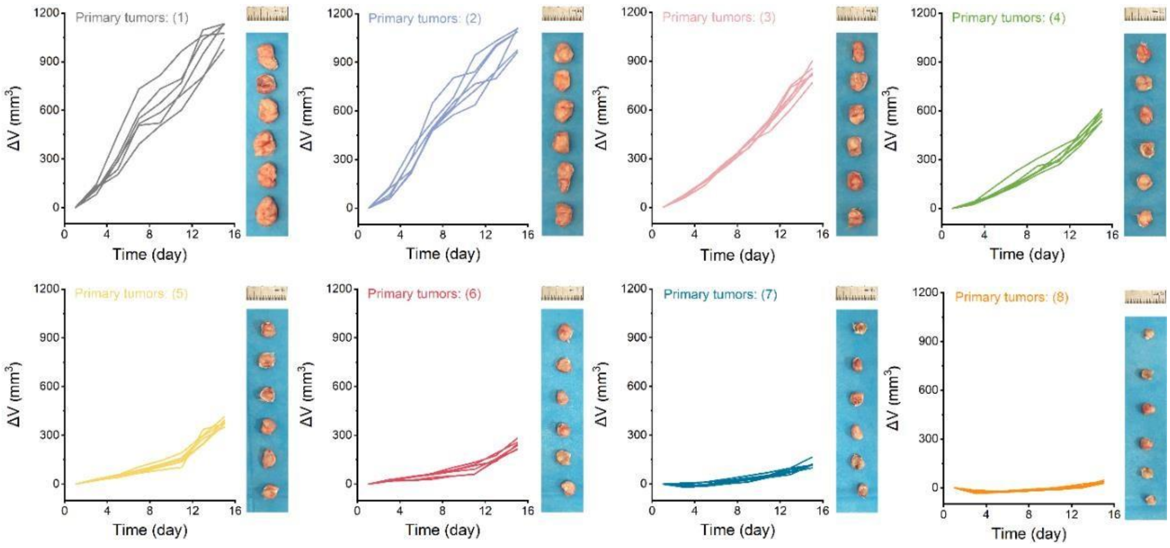

Fig. S32 Individual tumor growth curves of primary tumors after different treatments: (1) control, (2) $BaSO_4$, (3) αPD-1,(4) ZIF-8/TRF, (5) $BaSO_4$@ZIF-8, (6) $BaSO_4$@ZIF-8/TRF, (7) ZIF-8/TRF + αPD-1, and (8) $BaSO_4$@ZIF-8/TRF + αPD-1

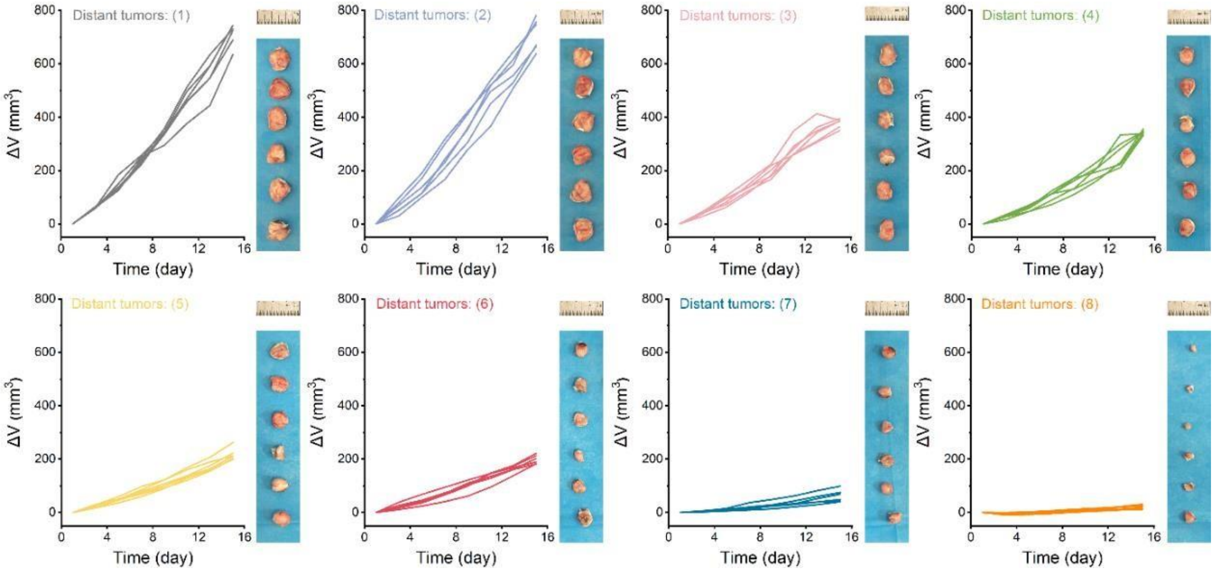

Fig. S33 Individual tumor growth curves of distant tumors after different treatments: (1) control, (2) $BaSO_4$, (3) αPD-1,(4) ZIF-8/TRF, (5) $BaSO_4$@ZIF-8, (6) $BaSO_4$@ZIF-8/TRF, (7) ZIF- 8/TRF + αPD-1, and (8) $BaSO_4$@ZIF-8/TRF + αPD-1

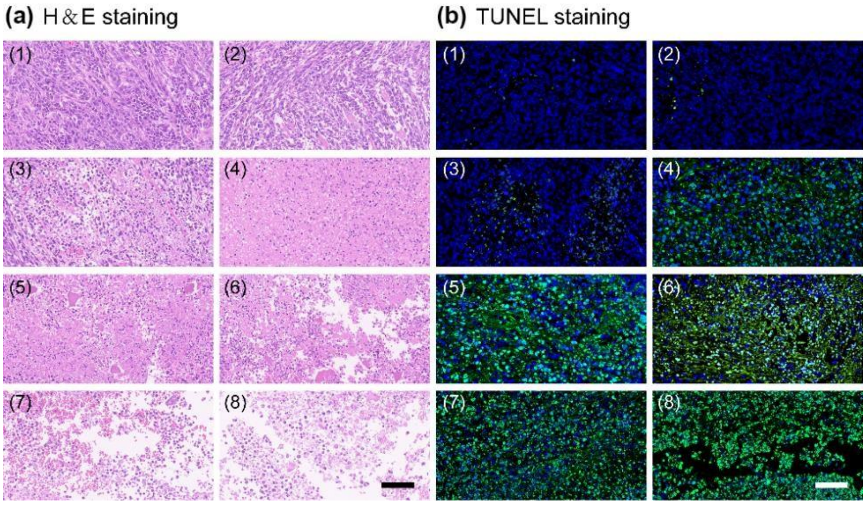

Fig. S34 (a) H&E staining and **(b)** TUNEL staining images of primary tumor slices in tumor- bearing mice after different treatments: (1) control, (2) $BaSO_4$, (3) αPD-1,(4) ZIF-8/TRF, (5) $BaSO_4$@ZIF-8, (6) $BaSO_4$@ZIF-8/TRF, (7) ZIF-8/TRF + αPD-1, and (8) $BaSO_4$@ZIF-8/TRF+ αPD-1. DNA fragmentations were stained by TUNEL (green fluorescence), and the nuclei were stained by Hoechst33342 (blue fluorescence). Scale bars = 50 μm

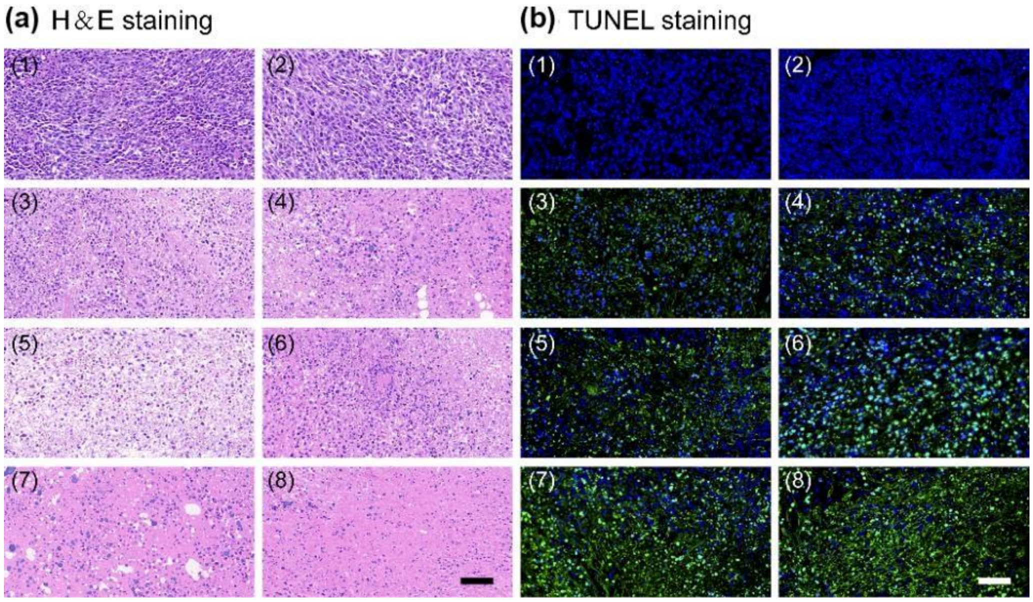

Fig. S35 (a) H&E staining, and **(b)** TUNEL staining images of distant tumor slices in tumor- bearing mice after different treatments: (1) control, (2) $BaSO_4$, (3) αPD-1,(4) ZIF-8/TRF, (5) $BaSO_4$@ZIF-8, (6) $BaSO_4$@ZIF-8/TRF, (7) ZIF-8/TRF + αPD-1, and (8) $BaSO_4$@ZIF-8/TRF+ αPD-1. DNA fragmentations were stained by TUNEL (green fluorescence), and the nuclei were stained by Hoechst33342 (blue fluorescence). Scale bars = 50 μm

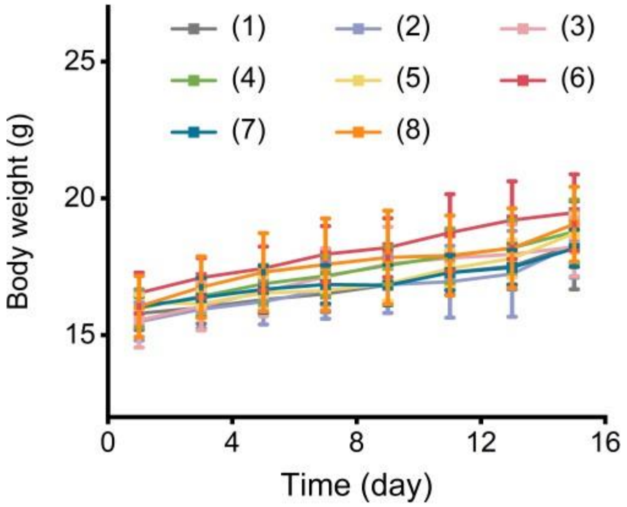

Fig. S36 Body weights of mice in various groups (n = 6 in each group): (1) control, (2) $BaSO_4$, (3) αPD-1, (4) ZIF-8/TRF, (5) $BaSO_4$@ZIF-8, (6) $BaSO_4$@ZIF-8/TRF, (7) ZIF-8/TRF + αPD-1, and (8) $BaSO_4$@ZIF-8/TRF + αPD-1

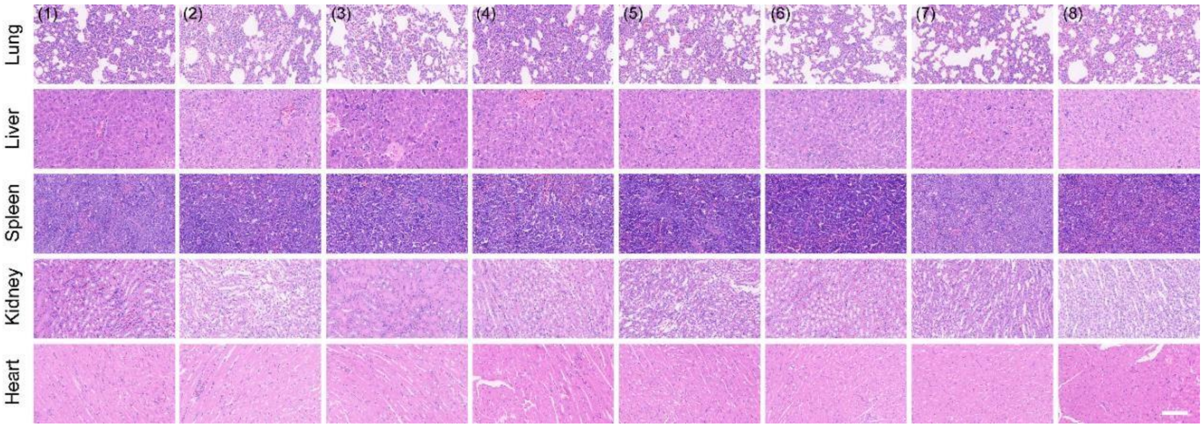

Fig. S37 H&E staining images of main organs in various groups: (1) control, (2) $BaSO_4$, (3) αPD-1,(4) ZIF-8/TRF, (5) $BaSO_4$@ZIF-8, (6) $BaSO_4$@ZIF-8/TRF, (7) ZIF-8/TRF + αPD-1, and (8) $BaSO_4$@ZIF-8/TRF + αPD-1. Scale bars = 100 μm

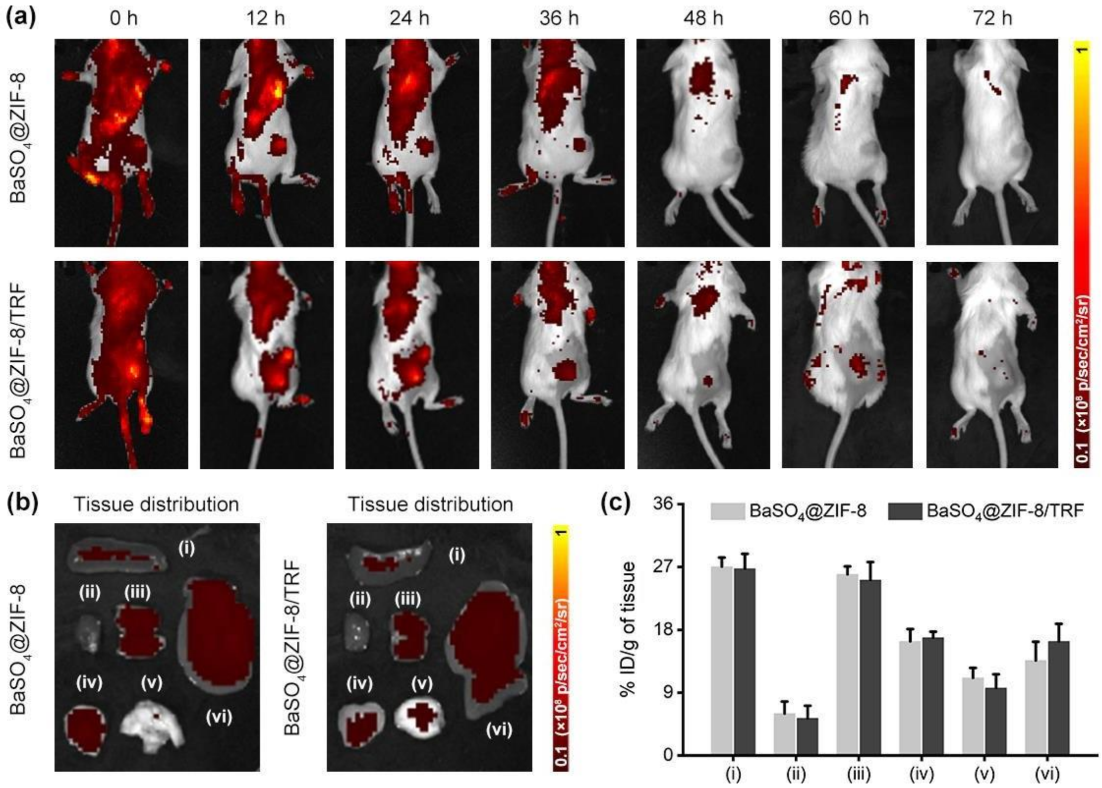

Fig. S38 (a) Near-infrared fluorescence images and **(b)** biodistribution results of $BaSO_4$@ZIF- 8 and $BaSO_4$@ZIF-8/TRF in 4T1 tumor-bearing mice post intravenous injection (i: spleen; ii: heart; iii: kidney; iv: tumor; v: lung; vi: liver). **(c)** Biodistributions of $BaSO_4$@ZIF-8 nanoparticles within the main organs of in 4T1 tumor-bearing mice at 72 h post intravenous injection

Table S1 Zeta potential of various nanomaterials

Nanomaterials	Zeta potential (mV)
$BaSO_4$	−36.6 ± 0.7
ZIF-8	34.6 ± 2.9
ZIF-8/TRF	−9.0 ± 1.1
$BaSO_4$@ZIF-8	−29.8 ± 0.6
$BaSO_4$@ZIF-8/TRF	−22.4 ± 0.9
